# Bioadhesive and hemostatic microneedles integrated with NIR-responsive Ti_3_C_2_/CeO_2_ heterojunction nanozymes for accelerated diabetic wound healing

**DOI:** 10.7150/thno.130627

**Published:** 2026-03-09

**Authors:** Zesheng Chen, Zhengyao Zhang, Lingfeng Tu, Guanyi Wang, Wang Wang, Ziqiang Xu, Feng Yu, Yinsuo Jia, Zijian Wang, Kanghong Hu, Weikang Hu

**Affiliations:** 1 Stem Cells and Tissue Engineering Manufacture Center, School of Life Sciences, Hubei University, Wuhan 430062, China.; 2 Hubei Province Key Laboratory of Biotechnology of Chinese Traditional Medicine, College of Health Science and Engineering, Hubei University, Wuhan 430062, China.; 3 Department of Urology, Hubei Key Laboratory of Urological Diseases, Cancer Precision Diagnosis and Treatment and Translational Medicine Hubei Engineering Research Center, Zhongnan Hospital of Wuhan University, Wuhan 430071, China.; 4 Department of Biomedical Engineering, Hubei Province Key Laboratory of Allergy and Immune Related Disease, TaiKang Medical School (School of Basic Medical Sciences), Wuhan University, Wuhan 430071, China.; 5 Department of Nuclear Medicine, Chongqing Hospital, Union Hospital, Tongji Medical College, Huazhong University of Science and Technology, 799 Liangjiang Avenue, Yubei District, Chongqing 401121, China.

**Keywords:** diabetic wound, heterojunction nanozyme, bilayer microneedles, tissue adhesion, NIR photocatalysis

## Abstract

**Rationale:**

The hyperglycemic microenvironment and microvascular dysfunction in diabetic wounds induce excessive reactive oxygen species (ROS) accumulation and persistent bleeding, severely impeding healing. Conventional nanozymes can scavenge ROS by mimicking the activities of superoxide dismutase (SOD) and catalase (CAT), but generally exhibit low catalytic efficiency and poor physiological stability. Fabricating nanozyme heterojunctions offers a viable approach to circumventing the aforementioned drawbacks.

**Methods:**

TC (Ti_3_C_2_/CeO_2_) heterojunctions were fabricated via an etching step followed by a solvothermal approach. The TC nanosheets were incorporated into a GelMA hydrogel to form the tip layer, while methacrylated dopamine (DMA) was integrated into the base layer for bio-adhesive functionality. A bilayer microneedle patch (GTM) was fabricated via a two-step photopolymerization process. The hemostatic capacity, photothermal conversion, and antioxidation properties were systematically examined *in vitro*. Meanwhile, animal experiments were executed utilizing a rabbit model of hepatic bleeding and a severe cutaneous defect model in diabetic Sprague-Dawley (SD) rats.

**Results:**

Under NIR irradiation, the TC heterojunction exhibited significantly enhanced antioxidant enzymatic activity, driven by synergistic interfacial charge transfer and photothermal effects. The bio-adhesive base layer achieved rapid hemostasis in a hepatic hemorrhage model, reducing blood loss from 6.51 g to 0.93 g. A highly accelerated wound healing process was observed in a diabetic rat model of full-thickness skin defects following treatment with GTM under NIR irradiation, achieving 99.89% wound closure by Day 20 while alleviating oxidative stress and promoting collagen deposition.

**Conclusion:**

This study developed a bifunctional wound repair platform by integrating a strongly adhesive microneedle base with NIR-responsive heterojunction nanozymes, providing novel insights for overcoming challenges in diabetic wound healing.

## Introduction

Clinically, wounds that fail to heal completely within 30 days of injury are generally defined as chronic or refractory wounds. Diabetic patients are frequently afflicted by chronic wounds, which represent a major and prevalent complication of the disease. If not properly managed, they can rapidly progress to severe tissue infections and, in critical cases, significantly increase the risk of amputation 1. The core pathological mechanism of delayed wound healing in diabetes lies in the oxidative stress damage and microvascular lesions induced by the hyperglycemic environment 2. The interplay of these factors drives overproduction of reactive oxygen species (ROS) at the wound site, disrupting redox homeostasis, which, in turn, intensifies chronic inflammation and suppresses new blood vessel formation 3-5. In addition, diabetic microvascular complications can also cause coagulation disorders, further delaying the normal healing process 6. Therefore, effective management of diabetic chronic wounds requires a dual synergistic intervention strategy: rebuilding the wound redox microenvironment while effectively controlling bleeding complications 7,8. In recent years, researchers have developed a variety of innovative wound dressings that combine rapid hemostasis and antioxidant functions 9-11. However, existing dressings generally face key challenges, including insufficient mechanical strength, rapid *in vivo* degradation, and difficulty sustaining antioxidant activity. These technical bottlenecks severely limit their clinical translation potential and actual therapeutic effects. Therefore, developing novel dressings that can synergistically deliver multiple therapeutic functions, such as hemostasis, antioxidant protection, and tissue regeneration, constitutes a high-priority challenge in the field of diabetic wound healing.

The body's tissues and cells contain a variety of endogenous antioxidant enzymes that can scavenge reactive oxygen species (ROS) through catalytic reactions and maintain redox balance. Among these enzymes, superoxide dismutase (SOD), catalase (CAT), and glutathione peroxidase (GPx) are the most representative 12. However, in pathological conditions such as chronic wounds, the excessive production of ROS often exceeds the endogenous antioxidant system's clearance capacity. To address this issue, exogenous supplementation with antioxidant enzymes has become an important strategy to enhance ROS clearance 13. Nevertheless, natural enzymes suffer from intrinsic drawbacks, including susceptibility to environmental fluctuations, limited biological half-life, and inadequate structural stability, which make them prone to loss of catalytic activity during *in vitro* storage and *in vivo* application, thereby severely restricting their clinical translation 14.

In response to the native shortcomings of enzymatic proteins, nanozymes, defined as nanoscale materials possessing intrinsic enzyme-like activity, have been progressively engineered. Accumulating evidence indicates that nanozymes can mimic the catalytic activities of several endogenous antioxidant enzymes, including superoxide dismutase (SOD), catalase (CAT), peroxidase (POD), and glucose oxidase (GOx), thereby demonstrating remarkable translational potential 15. Among various nanozyme candidates, cerium dioxide (CeO_2_) has demonstrated remarkable superiority in managing diseases associated with oxidative stress due to its inherent dual antioxidant activity (simultaneously mimicking SOD-like and CAT-like functions). This activity stems from the unique lattice structure of CeO_2_, where cerium ions can undergo reversible redox switching between two oxidation states, Ce^3+^ and Ce^4+^, along with the concurrent formation of oxygen vacancies. This regenerative catalysis enables CeO_2_ to continuously and efficiently scavenge reactive oxygen species (ROS), effectively overcoming the drawbacks of natural enzymes, such as rapid consumption and poor stability 16. However, single-structure CeO_2_ nanoparticles suffer from relatively low catalytic efficiency, instability in physiological environments, and a tendency to aggregate, all of which hinder their clinical translation. Therefore, synergistic structure-property regulation of CeO_2_ through surface chemical modification, lattice engineering (elemental doping), or the assembly of composite nanostructures is a key research direction for enhancing its therapeutic potential 17.

To further enhance the activity of nanozymes, recent studies have focused on near-infrared (NIR) photoexcitation strategies. Studies have shown that NIR excitation can effectively improve the enzyme-like functions of nanomaterials, including antioxidant activity, through mechanisms such as photothermal effects or hot-electron injection 18. Based on this principle, our research group previously reported a near-infrared responsive polydopamine/manganese dioxide (PDA/MnO_2_) composite material (PM) and elucidated the mechanism of action of NIR: the system harnesses the superior photothermal conversion capability of PDA to indirectly enhance the catalytic kinetics of MnO_2_, consequently elevating the overall enzymatic performance of the nanozyme. It is worth noting that the interfacial structure formed by PDA and MnO_2_ can also promote charge transfer, thereby further optimizing the material's catalytic performance 19. Inspired by this success, we modified CeO_2_ with NIR-responsive materials (such as PDA). This is expected to significantly enhance the antioxidant nanozyme activity by establishing a photothermal-catalytic synergistic mechanism, thereby overcoming its intrinsically low activity.

Ti_3_C_2_ (MXene) is a novel two-dimensional (2D) transition metal carbide nanomaterial, typically obtained by the selective etching of MAX phase precursors (e.g., Ti_3_AlC_2_). This material possesses a unique graphene-like layered structure and is rich in surface functional groups (-OH, -O, -F, etc.). As an outstanding near-infrared (NIR) photothermal material, Ti_3_C_2_ (MXene) exhibits highly efficient photothermal conversion across the 650-1350 nm spectral range, achieving a conversion efficiency approaching 100%. Such outstanding capability is largely ascribed to the high density of free electrons and the distinctive localized surface plasmon resonance (LSPR) phenomenon. Furthermore, Ti_3_C_2_ (MXene) integrates excellent biocompatibility, ultra-high specific surface area, outstanding electrical conductivity, and favorable dispersion stability in water, complemented by its controllable biodegradability and low cytotoxicity. Taken together, these favorable attributes greatly strengthen its translational viability, positioning it as an ideal candidate for a new generation of multifunctional nanobiomaterials whose potential applications in the biomedical field have been widely validated 20,21. Theoretically, the hybridization of Ti_3_C_2_/CeO_2_ can impart photo-enhanced catalytic activity to the composite material through the excellent near-infrared photothermal effect of MXene. Furthermore, Ti_3_C_2_ MXene, a 2D material with metallic properties, can interface with semiconducting CeO_2_ nanoparticles to form either Schottky or Type II heterojunctions. 22,23. The formation of this heterojunction establishes an internal electric field across the interface, which efficiently facilitates the spatial separation of photoinduced electron-hole pairs, minimizes charge carrier recombination, and expedites charge transfer 23. Furthermore, the resulting interfacial effect reduces the composite material's effective band gap, optimizes its band structure, and simultaneously enhances the activity of antioxidant nanozymes. The formation of the heterojunction structure can also further improve its SOD-like and CAT-like multi-enzyme catalytic performance through modulating the concentration of oxygen vacancies and the Ce^3+^/Ce^4+^ valence state ratio on the CeO_2_ surface. Compared to existing CeO_2_ modification strategies 24-26, the synergistic strategy of interface effect and near-infrared light enhancement developed in this study is expected to more effectively promote the Ce^3+^/Ce^4+^ redox cycle, thereby significantly enhancing the activity of nanozymes. These improvements may greatly enhance the translational potential of cerium dioxide for antioxidant stress therapy (such as chronic wound management in diabetes).

Hydrogel microneedle technology, as an innovative transdermal drug-delivery platform, has demonstrated significant advantages in complex wound-healing applications 19,27-29. Microneedles, with their micron-sized tips, can painlessly penetrate the epidermal barrier and deliver bioactive ingredients to the dermis. Furthermore, microneedles possess initial mechanical hemostatic capabilities; the local pressure effect generated during puncture helps achieve early hemostasis after wound healing 30. However, traditional microneedle systems often lack sufficient hemostatic barrier stability and fixation effectiveness in the face of challenges such as high exudate in diabetic wounds and leakage caused by vascular fragility. To address this, we propose integrating the natural adhesion molecule methacrylated dopamine (DMA) into the microneedle base layer. Leveraging DMA's excellent biocompatibility and strong interfacial adhesion, this method successfully constructs a composite microneedle system with rapid hemostasis and strong wet-state adhesion, ensuring prolonged durability of the dressing under harsh wound conditions.

To address the healing challenges posed by high oxidative stress and vascular fragility in diabetic wounds, this study developed an NIR-enhanced antioxidant nanozyme heterojunction hydrogel microneedle system (GTM). This bilayer system integrates two functional modules: (1) a hemostatic adhesive base—leveraging the mussel-inspired adhesive properties of DMA (methacrylated dopamine) to achieve rapid wet-tissue adhesion and physical wound closure; and (2) an NIR-synergistic catalytic tip-loaded with Ti_3_C_2_/CeO_2_ (TC) nanozyme heterojunction, which exploits interfacial charge transfer and the localized surface plasmon resonance (LSPR) effect of MXene under NIR irradiation to activate CeO_2_ catalytic sites (Ce^4+^ ↔ Ce^3+^ cycle), thereby significantly enhancing ROS scavenging capacity.

**Figure 1:**
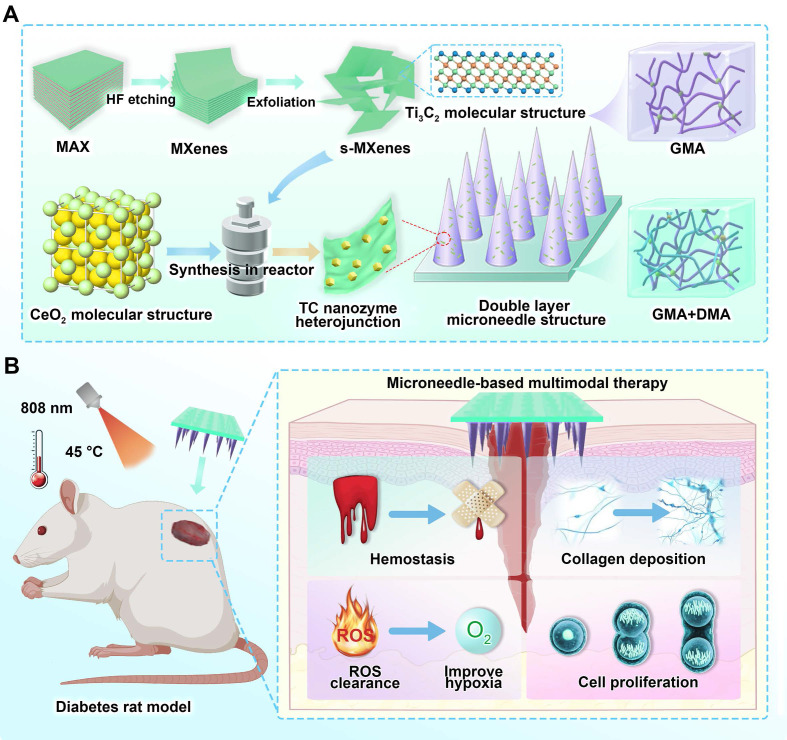
** Schematic illustration of the fabrication and application of bilayer microneedle patches incorporating Ti_3_C_2_/CeO_2_ (TC) nanozyme heterojunction and DMA bio-adhesive molecules.** (**A**) TC nanocomposites were synthesized via etching and solvothermal methods. TC was incorporated into the needle tips, while DMA was embedded within the base layer of the microneedles; (**B**) The bilayer microneedle system synergizes hemostatic capacity with anti-oxidant nanozyme activity, accelerating diabetic wound healing through ROS scavenging and enhanced collagen deposition.

**Figure 1** illustrates the overall design concept and fabrication workflow. A combined approach coupling selective etching with solvothermal treatment was employed to prepare the TC heterojunction. Benefiting from the excellent NIR photo-responsive properties of Ti_3_C_2_ and the interfacial synergistic effects, TC exhibited an enhanced antioxidant activity compared to pristine CeO_2_ 31, along with rapid photothermal heating and power-dependent temperature modulation, which are conducive to photothermal therapy and enhanced enzymatic catalysis. Subsequently, the bilayer microneedle system was fabricated through a two-step photopolymerization process: the basal layer consisted of GelMA-DMA composite hydrogel, achieving robust wet adhesion and rapid hemostasis superior to previously reported systems 32. Meanwhile, the tip layer comprised TC-loaded GelMA hydrogel for sustained NIR-enhanced antioxidant functionality.

After thoroughly evaluating the physicochemical characteristics, hemostatic performance, and ROS-scavenging ability of the system, its therapeutic efficacy was further validated *in vivo* through a full-thickness cutaneous wound model in diabetic SD rats. The findings revealed that the GTM system markedly facilitated wound closure, attenuated the hyperinflammatory response, and enhanced tissue regeneration.

In summary, the GTM bilayer microneedle system integrates rapid hemostasis, NIR-enhanced antioxidant activity, and photothermal therapeutic functions, with excellent biocompatibility, providing a novel, clinically viable approach for the targeted management of chronic diabetic wounds.

## Materials and Methods

### Materials

Bulk MXenes (Ti_3_AlC_2_) were purchased from XFNANO Tech. Co., Ltd (Jiangsu, China). Cerium nitrate (Ce(NO_3_)_3_·6H_2_O) was obtained from Sigma-Aldrich Co., Ltd (Shanghai, China). Methacrylated dopamine (DMA) was obtained from Yuan-Ye Biotech. Co., Ltd (Shanghai, China). Ammonia (NH_3_), hydrofluoric acid (HF), hydrochloric acid (HCl), lithium chloride (LiCl), absolute ethanol, hydrogen peroxide, and photoinitiator (Irgacure 2959) were purchased from Sinopharm Co., Ltd (Shanghai, China). Mouse lung L929 fibroblasts were kindly provided by the Medical Research Center, Zhongnan Hospital of Wuhan University. RPMI-1640 medium, fetal bovine serum, trypsin, and phosphate-buffered saline (PBS) were obtained from Thermo Fisher Scientific Co., Ltd (Shanghai, China). A gelatin-based hemostatic sponge was purchased from Kuai-Kang Co., Ltd (Guangzhou, China). Commercial wound dressings were purchased from 3M Co., Ltd (Shanghai, China).

### Preparation of monolayer Ti_3_C_2_ MXene nanosheets

A modified chemical etching method was applied to prepare the monolayer Ti_3_C_2_ nanosheets. Briefly, 1 g of bulk MXenes, 20 mL of HF acid, 20 mL of HCl solution, and 10 mL of H_2_O were mixed and then reacted at 37 ℃ for at least 12 h. Subsequently, the suspension was centrifuged; the supernatant was discarded, and the sediment was rinsed three times with distilled water. The obtained products were transferred into a 2% LiCl solution and stirred continuously for at least 12 h. After dispersion by ultrasonication for 30 min, Ti_3_C_2_ nanosheets with a monolayer structure were obtained.

### Preparation of Ti_3_C_2_/CeO_2_ (TC) nanozyme heterojunction

The Ti_3_C_2_/CeO_2_ (TC) nanozyme heterojunction was prepared using a hydrothermal reaction. Briefly, 0.5 g of Ce(NO_3_)_3_·6H_2_O was completely dissolved in 20 mL of distilled water. Next, 0.1 g of monolayer Ti_3_C_2_ nanosheets and 2 mL of ammonia solution were added to the precursor mixture, followed by ultrasonic dispersion for 30 min. The resulting suspension was then maintained at 180 °C for 24 h. The product (TC) was harvested by centrifugation (5000 rpm, 10 min), washed three times with distilled water, and lyophilized for subsequent characterization.

### Preparation of GelMA/TC heterojunction composite hydrogels

The synthesis and structural verification of GelMA were conducted according to established protocols described in our earlier work 33. GelMA was dissolved in distilled water to yield a 15 wt% solution. Subsequently, a defined amount of TC heterojunction, along with an optimized concentration of Irgacure 2959 (I2959) photo-initiator, was dispersed into the system and mixed uniformly for 30 min. Residual air bubbles were removed by centrifugation, and the precursor solution was then crosslinked by irradiating with 160 W UV light at 365 nm for 300 s. The resulting GelMA-TC heterojunction composite hydrogels were designated as GT-n, where n (1, 2, or 3) corresponds to the TC heterojunction content. Neat GelMA hydrogel (termed as GM) and GelMA-Ti_3_C_2_ hydrogel (termed as GTiC) were also prepared following the same protocol as controls. The sample codes and compositions are summarized in **Table 1**.

### Preparation of tissue-adhesive GelMA-DMA composite hydrogels

In this study, tissue-adhesive GelMA-DMA composite hydrogels were prepared as described in a previous report 34. DMA was introduced into distilled water under stirring to generate a 10% DMA solution. The GelMA solution, DMA solution, and photoinitiator were mixed by mechanical stirring for 30 min, then debubbled by centrifugation. These mixtures were transferred to a mold and irradiated with 160 W of UV light for 180 s. The resulting GelMA-DMA composite hydrogels were designated as GD-n, where n (0, 1, 2, or 3) corresponds to the DMA content. The sample designations and compositions are summarized in** Table 2**.

### Preparation of bilayer hydrogel microneedles

The microneedle arrays were prepared via a sequential two-step molding process. The polydimethylsiloxane (PDMS) molds were kindly provided by Xinyun Nanotech. Co., Ltd (Suzhou, China). Briefly, the GT-2 precursor solution was dispensed into the mold cavities, and entrapped air bubbles were removed under vacuum. The mold was then irradiated with UV light for 5 min to cure the tip layer. Subsequently, the GD-2 precursor solution was added onto the cured tips, followed by UV irradiation for 3 min to form the base layer. The resulting bilayer hydrogel microneedles were dried at room temperature, demolded, and designated as GTM microneedles (GT components form the needle tip, including GelMA and TC; GD components form the base, including GelMA and DMA). For comparison, GMM (neat GelMA microneedle), with both the needle tip and base composed of GelMA, was also prepared.

### Physicochemical characterization

Surface morphology and elemental mapping were acquired utilizing scanning electron microscopy (AMBER, TESCAN, Czech) and energy-dispersive X-ray spectroscopy (ChemiSEM, Thermo-Fisher, USA). The crystal lattice properties and chemical states of the TC heterojunction were determined via X-ray diffraction (Bruker D8 Advance, USA) and X-ray photoelectron spectroscopy (Thermo Fisher K-Alpha, USA). Finally, the light absorption capabilities were evaluated using a UV-3600 spectrophotometer (SHIMADZU, Japan). To assess the electron-hole pair recombination kinetics, the samples were subjected to steady-state and time-resolved photoluminescence (PL) spectroscopy utilizing an Edinburgh FLS1000 spectrometer. Details regarding other physicochemical characterizations are provided in the Supplementary Information.

The kinetic parameters of CeO_2_ and TC were determined with different concentrations of H_2_O_2_ (0, 5, 10, 15, 20, 30, 50, 80, and 100 mM) and riboflavin solutions (0, 0.05, 0.1, 0.2, 0.3, 0.4, 0.6, 0.8, and 1 mM) as substrates under optimal reaction conditions. The Michaelis-Menten equation was applied to determine the maximum catalytic velocity (V_max_) and the Michaelis constant (K_m_).

All density functional theory (DFT) computations were carried out within the generalized gradient approximation (GGA) framework, employing the Perdew-Burke-Ernzerhof (PBE) exchange-correlation functional. The ionic cores were described using projected augmented wave (PAW) pseudopotentials, while the valence electronic states were expanded in a plane-wave basis set with an energy cutoff of 450 eV. The Gaussian smearing technique with a smearing width of 0.05 eV was adopted to handle the partial occupancies of Kohn-Sham orbitals. Structural and lattice parameter optimizations were conducted with Brillouin zone sampling using a Γ-centered Monkhorst-Pack k-point mesh at a resolution of 0.004 Å^-1^. The self-consistent calculations applied a convergence energy threshold of 10^-5^ eV. The equilibrium geometries and lattice constants were optimized such that the maximum stress on each atom was within 0.02 eV Å^-1^. A vacuum spacing of 15 Å was introduced along the surface normal direction to prevent spurious interactions between neighboring periodic replicas of the heterostructure. Weak van der Waals forces were accounted for through the DFT-D3 approach incorporating Grimme's empirical dispersion correction. Spin-polarized calculations were employed to properly capture the magnetic properties of the system. To address the strong electron correlation effects inherent in transition metal elements, both geometric optimization and electronic structure analysis were conducted within the spin-dependent GGA+U framework, where the effective Hubbard Ueff values were set to 4.0 eV and 5.0 eV for Ti and Ce atoms, respectively.

### Photocatalytic tests

The CeO_2_ and TC powders were homogeneously dispersed in an aqueous solvent to prepare a 5 mg/mL solution. The solution was uniformly drop-cast onto conductive carbon paper and dried at 60 °C. This coating process was repeated three times to fabricate the working electrode. Electrochemical measurements were conducted in a standard three-electrode configuration, employing an Ag/AgCl electrode as the reference and a platinum electrode as the counter electrode, and the material-coated carbon paper as the working electrode, with 5 % sodium sulfate as the electrolyte. Both electrochemical impedance spectroscopy (EIS) and linear sweep voltammetry (LSV) measurements were performed at a fixed applied potential of 0.5 V. For the photoelectrochemical evaluation, the working electrode was irradiated with near-infrared (NIR) light (2.5 W/cm²) for 30 s, with a subsequent 30 s dark period forming a single on-off cycle. The photocurrent response and cyclic durability of the material were then systematically evaluated across multiple successive cycles.

### Photothermal testing

The photothermal properties of Ti_3_C_2_ nanosheets and TC heterojunctions were evaluated under 808 nm NIR laser irradiation at a power density of 2.5 W/cm^2^. Pure water without nanoparticles served as the blank control. For the nanoparticle-laden hydrogels, considering the light scattering and attenuation caused by the hydrogel matrix, a slightly higher power density (3.0 W/cm^2^) was applied to the microneedle system compared to the single Ti_3_C_2_ nanosheets and TC heterojunctions (2.5 W/cm^2^), and then the GM, GTiC, and GT hydrogels were cut into small pieces and subjected to NIR irradiation (808 nm, 3.0 W/cm^2^) for 180 s. Real-time thermal imaging was captured using an infrared camera (Fortric 223s, China) throughout the irradiation process. To evaluate the power-dependent photothermal performance, GT hydrogel was irradiated at varied power densities of 1.5, 2.0, 2.5, and 3.0 W/cm^2^. For cyclic photothermal stability testing, the GT hydrogel was subjected to NIR irradiation (3.0 W/cm^2^) for 60 s per cycle, followed by natural cooling to room temperature before the subsequent irradiation. This process was repeated for six consecutive cycles. For *in vivo* photothermal experiments, eight female SD rats weighing 200 g were obtained from Shulaibao Co., Ltd (Wuhan, China). The samples were attached to the backs of the mice and irradiated at a power density of 3.0 W/cm^2^.

To further evaluate the photothermal conversion efficiency of TC, we prepared a 500 μg/mL TC solution in ultrapure water and exposed it to an 808 nm NIR laser at a power density of 2.5 W/cm^2^ to assess its photothermal performance according to the method reported in the literature 35. The temperature variation of the TC solution under natural cooling was also recorded. The photothermal conversion efficiency (η) was calculated using the following formula:

τs= 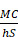
(1)

Q= hS (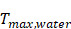-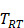) (2)

η= 
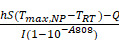
(3)

In the fitting calculations, τs is the time constant. M, C, and S represent the mass of water, specific heat capacity, and surface area of the container, respectively. h is the heat transfer coefficient of the reaction system. T_max_ and T_RT_ indicate the highest temperature of solution and ambient temperature, respectively. Q represents the baseline energy generated by 808 nm laser irradiation, I is the power density of the laser, and A808 indicates the absorbance at 808 nm of TC.

### Anti-oxidant nanozyme activity test

CeO_2_, Ti_3_C_2_, and TC were aliquoted into separate test tubes containing the following reaction reagents: methionine solution (Met), nitroblue tetrazolium (NBT), EDTA-Na, and riboflavin. Following NIR irradiation (2.5 W/cm^2^) for 180 s, the samples were immediately transferred to darkness to terminate the reaction. Superoxide dismutase (SOD)-mimetic activity was quantified by measuring the absorbance at 560 nm using a UV-vis spectrophotometer.

Parallel experimental setups were prepared with CeO_2_, Ti_3_C_2_, and TC in hydrogen peroxide (H_2_O_2_) solutions. After identical NIR exposure parameters (2.5 W/cm^2^, 180 s), catalase (CAT)-mimetic activity was determined through absorbance measurements at 240 nm.

EPR (Electron paramagnetic resonance) for •O_2_^-^ detection: methanol (100 μL), xanthine oxidase and xanthine were mixed and reacted for 5 min, followed with the addition of TC and CeO_2_ dispersions. After another 5 min, DMPO (250 mM) was added as a trapping agent. The solution was immediately transferred to a sample tube to record the EPR spectrum of •O_2_^-^.

EPR for ·OH detection, double distilled water (ddH_2_O, 50 μL), H_2_O_2_ (10 mM, 50 μL), and FeCl_2_ (1 mM, 20 μL) were mixed and reacted for 5 min, followed by the addition of TC and CeO_2_ dispersions. After another 5 min, DMPO (250 mM) was added as a trapping agent. The resulting solution was promptly transferred into a sample tube for the acquisition of the ·OH electron paramagnetic resonance (EPR) spectrum.

To systematically investigate the anti-oxidant capacity, microneedles (MNs) were tested under NIR power densities at 3.0 W/cm^2^, different composite material concentrations (0-10 mg/mL), and different NIR exposure times (0-20 min). All measurements were conducted in triplicate with strict light-control protocols.

The maximum initial reaction rate (V_max_), Michaelis constant (K_m_), and catalytic constant (K_cat_) of the enzymes were calculated by fitting the data to the Lambert-Beer law, the rate equation, the Michaelis-Menten equation, and the Lineweaver-Burk equation.

### Hemostatic tests

This study was approved by the Animal Ethics and Welfare Committee of Hubei University (Certificate No. 20250003). Twelve female New Zealand rabbits, weighting approximately 2.5 kg, were kindly obtained from Shulaibao Co., Ltd (Wuhan, China). A liver bleeding model was established to evaluate the hemostatic effect of the GD-2 hydrogel. Following anesthesia with inhaled isoflurane, all animals were randomly allocated into four groups: B.C. (Blank control), GD-0 (also called GM), P.C. (Positive control), and GD-2. They were safely fixed on surgical corkboards, and their abdomens were cut to expose the liver. After that, a 10 mm long surgical wound was made in each liver. The wounds in the GD-2 and GD-0 groups were immediately covered with a piece of GD-2 or GD-0 hydrogel, and those in the P.C. group were treated with a gelatin-based hemostatic sponge. The B.C. group was not treated. The bleeding time and blood loss were recorded. Fresh liver samples were resected for H&E staining analysis.

### Biocompatibility and anti-oxidative evaluations

This study was approved by the Animal Ethics and Welfare Committee of Hubei University (Certificate No. 20250003). *In vivo* biocompatibility was assessed through a subcutaneous implantation model using Sprague-Dawley (SD) rats. Twelve female SD rats with a body weight of approximately 200 g were purchased from Shulaibao Co., Ltd (Wuhan, China). These animals were safely anesthetized and then subcutaneously implanted with GD-0 (also called GM), GD-2, and GT hydrogels for 14 days. The B.C. group underwent a sham operation. After that, fresh plasma was collected at a clinical laboratory for a series of blood biochemical tests. Fresh tissues (heart, liver, spleen, lung, kidney, and brain), the hydrogels, and the surrounding capsules were also collected for H&E staining analysis. Data acquisition was performed using a laser confocal microscope (LCS-SP8-STED, Leica, Germany). The cellular responses under oxidative conditions, evaluated via flow cytometry (L929) and transwell assays (L929 and HUVECs), are detailed in the Supplementary Information.

### Wound healing tests

This study was approved by the Animal Ethics and Welfare Committee of Hubei University (Certificate No. 20250003). To assess the wound healing-promoting capability of GTM microneedles, a full-thickness cutaneous wound model was constructed in diabetic SD rats. Twenty-five female SD rats with type II diabetes were obtained from Shulaibao Co., Ltd (Wuhan, China). After anesthesia, the dorsal hair was removed entirely, and a 15 × 15 mm^2^ full-thickness skin defect was created on each rat. All animals were randomly allocated into five experimental groups, with five rats in each group (n = 5 per group): (1) blank control (B.C.) group, which received no treatment, (2) positive control group treated with 3M (3M™ Tegaderm™ Hydrocolloid Thin dressing; Model: 90022T) commercial dressing, (3) GMM group treated with neat GelMA microneedles, (4) GTM group treated with GelMA microneedles containing TC-loaded tips and DMA-incorporated base, and (5) GTM/N group treated with GTM microneedles combined with NIR-mediated photothermal therapy (808 nm, 3.0 W/cm², 3 min). All experimental animals were maintained under specific-pathogen-free (SPF) conditions for a period of 20 days. To evaluate the general health and systemic biosafety of the treatments, the body weights of the diabetic rats were monitored at predetermined time intervals (days 0, 5, 10, 15, and 20) using an electronic balance. Photographs of the wound area were taken at designated time points (days 0, 5, 10, 15, and 20). The newly formed skin tissues were then excised for histological examination.

### Statistical analysis

Quantitative results are reported as the mean ± standard deviation (SD). For multiple group comparisons, statistical significance was determined utilizing a one-way analysis of variance (ANOVA) in conjunction with a Tukey's post-hoc test. Significance levels were set as follows: n.s. indicates no significance, *P < 0.05, **P < 0.01, ***P < 0.001.

## Results and Discussion

### Fabrication and characterization of the TC nanosheets

**Figure 2:**
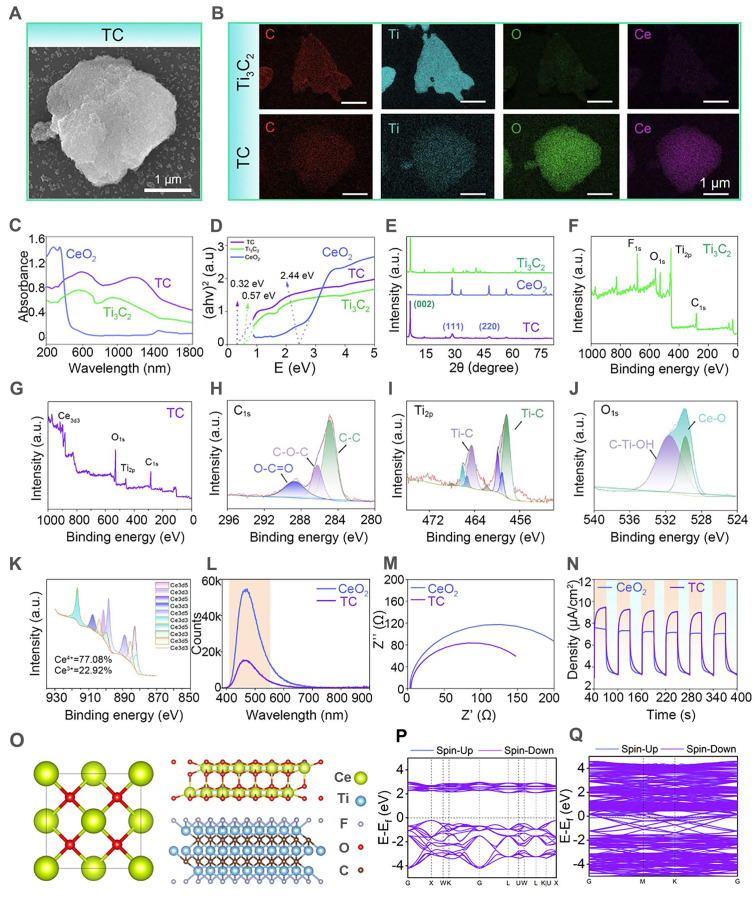
**Preparation and characterization of the Ti_3_C_2_/CeO_2_ (TC) heterojunction.** (**A**) SEM images of the TC heterojunction. Scale bar: 1 µm; (**B**) SEM map of Ti_3_C_2_ nanosheets and the TC heterojunction. Scale bar: 1 µm; (**C**) UV-Vis spectrum; (**D**) The bandgap calculated by the Tauc-Plot method; (**E**) XRD spectrum; (**F**) XPS spectrum of the Ti_3_C_2_ nanosheets; (**G**) XPS spectrum of the TC heterojunction; (**H**) C 1s XPS spectrum of the TC heterojunction; (**I**) Ti 2p XPS spectrum of the TC heterojunction; (**J**) O 1s XPS spectrum of the TC heterojunction; (**K**) Ce XPS spectrum of the TC heterojunction; (**L**) Photoluminescence spectrum; (**M**) AC impedance spectrum; (**N**) i-t curve; (**O**) Theoretical models of CeO_2_ and TC; (**P** and** Q**) DFT analysis of the band gaps of CeO_2_ and TC.

In this study, Ti_3_C_2_ and TC nanosheets were synthesized through etching and solvothermal methods. As shown in **Figure S1**, the initial Ti_3_AlC_2_ precursor exhibited a bulk morphology. After sequential HF etching and LiCl intercalation, monolayer Ti_3_C_2_ nanosheets with well-defined lamellar structures and uniform sheet geometry were successfully obtained. **Figure 2A** and **Figure S2** display the SEM (Scanning electron microscope) micrographs of the TC nanosheets, demonstrating the uniform growth of CeO_2_ nanoparticles on the Ti_3_C_2_ surface in the form of 20 nm microspheres via a solvothermal reaction. The composite structure was further confirmed by energy-dispersive X-ray spectroscopy (EDS), as shown in **Figure 2B**. EDS elemental mapping revealed distinct compositional differences: pristine Ti_3_C_2_ showed exclusive Ti and C signals, whereas the TC nanosheets presented predominant Ce and O signals from the surface-anchored CeO_2_, accompanied by attenuated Ti and C intensities due to adequate CeO_2_ coverage.

The chemical composition and structural properties of TC were further investigated using UV-vis diffuse reflectance spectroscopy (DRS), X-ray diffraction (XRD), and X-ray photoelectron spectroscopy (XPS). As revealed by the UV‒vis DRS spectra (**Figure 2C**) and derived bandgap calculations (**Figure 2D**), the TC composite demonstrated significantly enhanced near-infrared (NIR) light absorption compared with those of pristine CeO_2_ and Ti_3_C_2_. Notably, interfacial effects arising from the TC nanosheets reduced the bandgap of TC to 0.32 eV, a substantial decrease from CeO_2_'s original value of 2.44 eV. This unique interface effect enabled more electrons to be excited into the conduction band by near-infrared light. The reduced band gap facilitated enhanced electron-hole pair generation, contributing to the improved catalytic efficiency of the composite.

In the XRD pattern (**Figure 2E**), the Ti_3_C_2_ nanoparticles presented characteristic peaks at 8.5° (0 0 2) 36; The CeO_2_ nanoparticles presented characteristic peaks at 28.5° (1 1 1), 33° (2 0 0), 47.5° (2 2 0), and 56° (3 1 1) 37. For the TC heterojunction, peaks at 8.5°, 28.5°, and 33° were observed, suggesting that the TC nanosheets were composed of Ti_3_C_2_ and CeO_2_.

**Figure 2F** shows that Ti_3_C_2_ primarily exhibited characteristic peaks of Ti 2p and C 1s. Additionally, owing to the abundant functional groups introduced on the material surface during the etching reaction, minor O 1s and F 1s characteristic peaks were observed on the Ti_3_C_2_ surface. In the XPS spectrum of the TC (**Figure 2G**), the characteristic Ce 3d peak was detected in the curve, confirming the successful growth of CeO_2_ on the Ti_3_C_2_ surface. To further investigate the chemical bonding states of the material, high-resolution XPS elemental spectra of TC were analyzed (**Figure 2H-K**). The C 1s spectrum displayed three main characteristic peaks at 284.8 eV (C-C), 285.5 eV (C-O-C), and 289 eV (O-C=O) 38. These peaks indicated the presence of diverse carbon bonding states on the composite surface, resulting from oxidation reactions with air during the etching process. The Ti 2p spectrum showed characteristic Ti-C peaks, indicating the presence of well-preserved Ti_3_C_2_ in the composite. The Ce elemental spectrum showed characteristic peaks at 531.5 eV (Ce-O) and 533.1 eV (C-Ti-OH) 39, corresponding to cerium oxide and hydroxylated titanium species, respectively. The Ce-O peak further verified the successful incorporation of CeO_2_. The Ce elemental spectrum revealed the coexistence of Ce^3+^ and Ce^4+^ in the TC material. The peaks at 885, 898, and 902 eV belong to Ce^3+^, whereas those at 882, 888, 898, 900, 907, and 916 eV belong to Ce^4+^ 40. The presence of Ce^3+^/Ce^4+^ redox pairs endowed TC with enhanced electron-transfer capability during photocatalytic processes, thereby facilitating improved catalytic performance 41. The above characterization collectively verified that the TC heterojunction was properly synthesized with the expected composition and structure.

### Evaluation of the photocatalytic performance

In this study, to enhance the anti-oxidant nanozyme activity of CeO_2_, we introduced the two-dimensional MXene material Ti_3_C_2_, which has excellent electrical conductivity and NIR photocatalytic properties. Owing to the photocatalytic capability of MXenes and the unique interfacial effects between CeO_2_ and Ti_3_C_2_, the TC composite demonstrated superior photogenerated electron-hole pair generation and electron transfer efficiency, thereby significantly enhancing its anti-oxidant nanozyme activity. The photocatalytic activity of TC was further assessed through photoluminescence (PL) spectral analysis and a series of electrochemical characterizations.

As shown in the PL spectrum (**Figure 2L**), compared to unmodified CeO_2_, the TC composite exhibits a substantially diminished photoluminescence signal, providing direct evidence that the recombination of photogenerated electron-hole pairs has been effectively suppressed. This suggests that more photogenerated carriers participate in the Ce^3+^/Ce^4+^ redox cycle, directly revealing the enhanced anti-oxidant nanozyme activity of TC 42. This enhancement is largely ascribed to rational material engineering and strategic structural optimization. Electrochemical tests further corroborated these findings. Electrochemical impedance spectroscopy (**Figure 2M**) revealed a substantially reduced impedance value for TC, owing to the outstanding conductivity of Ti_3_C_2_, which effectively reduced the energy dissipation associated with photoinduced charge carrier transport within the material. Concurrently, the photocurrent-time (i-t) curve (**Figure 2N**) demonstrated a higher photogenerated carrier density in the TC under NIR irradiation 43. These experimental results collectively verify the successful modification of TC, emphasizing its significant potential as a highly promising material for antioxidant stress applications.

DFT simulations were further employed to corroborate the changes in the material's band structure. As shown in **Figure 2O**, we constructed models of CeO_2_ and TC materials, and subsequently calculated their band structures (**Figures 2P** and **2Q**). The computational results reveal that CeO_2_ displayed a characteristic semiconductor electronic structure with a band gap of 2.32 eV, in good agreement with the experimentally determined value. It is worth noting that the PBE functional employed in our simulations tends to slightly underestimate the band gap energy 44. For the TC material, owing to the metallic character imparted by Ti_3_C_2_ 45,46, the Fermi level crossed the bands in its band structure, confirming its metallic nature. By coupling this semiconductor with a metallic component to construct a Schottky heterojunction, the transport and dissociation of photoexcited charge carriers were effectively promoted, leading to a remarkable improvement in antioxidant nanozyme performance 47,48.

In summary, the band structure and electron transfer mechanism of TC, a Schottky heterojunction, can be elucidated as follows (**Figure S3**). Under NIR light excitation, photogenerated electrons from the conduction band of CeO_2_ rapidly transfer to the surface of Ti_3_C_2_ due to its lower work function. This process effectively suppresses the recombination of photogenerated electron-hole pairs. This not only enhances the photothermal conversion efficiency but, more importantly, promotes the accumulation of holes on the CeO_2_ surface, thereby strengthening the activity of its redox cycle (Ce^3+^/Ce^4+^). As a result, it synergistically and significantly improves the reactive oxygen species (ROS) scavenging capability of nanozyme-like enzymes, particularly CAT and SOD. This paves the way for the subsequent engineering of microneedle patches endowed with antioxidant properties.

### Characterization of the tip layer of the hydrogel microneedles

**Figure 3:**
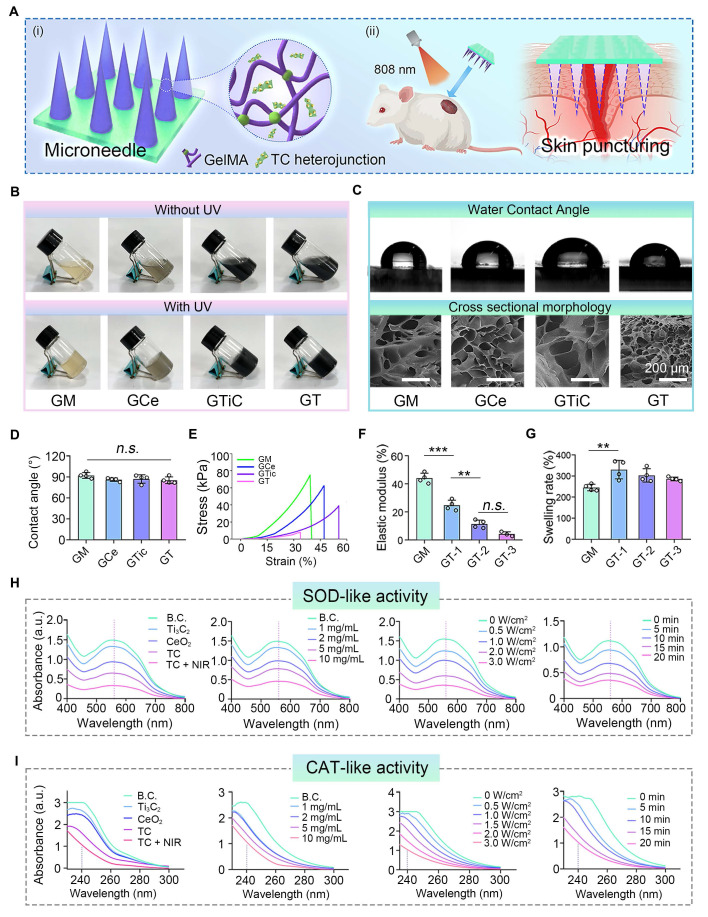
** Preparation and characterization of the tip layer of the hydrogel microneedles.** (**A**) Schematic illustration showing the intended microneedle application, with the experiments in this figure focusing on the fundamental characterization of TC nanosheets in the hydrogel matrix: (i) TC is incorporated into the molecular network of the GelMA hydrogel; (ii) the tip layer can puncture skin tissue for NIR-assisted treatment; (**B**) Optical images of the hydrogels before and after UV crosslinking (GM: pure GelMA hydrogel; Gce: GelMA-CeO_2_ hydrogel; GTiC: GelMA-Ti_3_C_2_ hydrogel; GT: GelMA-TC hydrogel); (**C**) Images of the water contact angle and cross-sectional morphology of freeze-dried hydrogels. Scale bar: 200 µm; (**D**) Quantitative results of water contact angle (n = 4); (**E** and **F**) Compression test (n = 4); (**G**) Equilibrium swelling rate (n = 4); (**H**) UV-vis spectrophotometry results of SOD-like enzyme activity; **(I)** UV-vis spectrophotometry results of CAT-like enzyme activity. Values are expressed as the means ± SD. For group comparisons, *n.s.* indicates no significance, **P < 0.01, ***P < 0.001.

To endow microneedles with robust anti-oxidant stress resistance, we loaded TC with anti-oxidative nanozyme activity into the microneedle tip layer based on GelMA (**Figure 3A**). To systematically evaluate the impact of material incorporation on the physicochemical properties of the hydrogel tip layer, composite hydrogels with different additives were prepared for comparative analysis. As shown in **Figure 3B**, the pure GelMA solution appeared pale yellow, whereas color variations were observed upon the addition of CeO_2_, Ti_3_C_2_, and TC. All the precursor solutions could be able to form hydrogels after UV-induced photopolymerization (GM: pure GelMA hydrogel; Gce: GelMA-CeO_2_ hydrogel; GTiC: GelMA-Ti_3_C_2_ hydrogel; GT: GelMA-TC hydrogel). Notably, the GT composite hydrogel underwent a UV-mediated chemical crosslinking transition from the liquid state to the solid state under ultraviolet irradiation, confirming that TC maintained good dispersibility in GelMA without significantly compromising its gelation capability.

The cross-sectional morphology and water contact angle data of the hydrogels are presented in **Figure 3C** and** D**. The freeze-dried hydrogels displayed well-defined porous architectures that enabled effective uptake of wound exudate while sustaining a moist healing environment. Water contact angle analysis confirmed that the introduction of additional materials preserved the intrinsic hydrophilic nature of the hydrogel substrate. Given the mechanical stresses exerted on skin during movement, biomedical dressings require appropriate elastic properties. To verify the structural robustness of the hydrogels, their mechanical response to compressive loading was systematically evaluated. As illustrated in **Figures 3E** and **F**, although additive incorporation slightly reduced the compressive strength due to the modified network architecture, all hydrogel formulations satisfied the mechanical performance criteria necessary for biomedical use, providing critical support for the subsequent fabrication of mechanically robust microneedle patches.

The swelling properties were further investigated (**Figure 3G**). Upon 24 h of immersion, the hydrogels attained their swelling equilibrium state. As the degree of crosslinking within the polymer network increased, the equilibrium water uptake capacity correspondingly declined 49. The introduction of additional components partially reduced the crosslinking degree of GelMA hydrogels, thereby enhancing their water absorption capability.

### Evaluation of anti-oxidative stress performance

Excessive ROS in hyperinflammatory responses exacerbate local tissue damage, leading to chronic inflammation and severely impeding diabetic wound healing 50,51. Advances in nanomedicine have provided innovative strategies for ROS scavenging, and the development of nanozymes to maintain the natural redox balance in biological systems has emerged as a promising approach 52. Ideal nanozymes should demonstrate ROS-scavenging capacity comparable to that of natural enzymes (e.g., CAT and SOD) 53. We therefore systematically evaluated the anti-oxidant performances of the TC-loaded hydrogels. As demonstrated in** Figure 3H** and **I**, the TC composite exhibited superior SOD-like and CAT-like nanozyme activities, with the enzymatic performance slightly surpassing that of pure CeO_2_. This enhancement could be attributed to the interfacial engineering between CeO_2_ and Ti_3_C_2_ in TC, which effectively separated the photogenerated electron-hole pairs required for catalytic reactions, thereby amplifying the nanozyme-mimetic functionality 54. Comparative analysis further revealed intensified nanozyme-like activities under near-infrared (NIR) irradiation, where the photothermal effect generated additional charge carriers to ultimately boost catalytic efficiency 55. Furthermore, the SOD-like and CAT-like activities of TC showed concentration-dependent responses to H_2_O_2_, NIR power intensity, and irradiation duration. These parametric studies confirmed the precisely tunable antioxidant behavior of TC.

The EPR test results (**Figure S4**) further validate this conclusion. Compared to pure CeO_2_, the EPR signals for •OH and•O_2_^-^ were significantly weakened in the presence of the TC material, indicating a notable enhancement in its radical scavenging capability. This direct experimental evidence strongly confirms the substantial improvement in antioxidant performance achieved through the construction of the Ti_3_C_2_/CeO_2_ heterojunction.

To further evaluate the nanozyme activity of the composite material, we measured the steady-state kinetic curves of CeO_2_ and TC using H_2_O_2_ and riboflavin as substrates (**Figure S5**). Experimental results demonstrated that the CAT-like and SOD-like mimicking catalytic rates of both materials progressively reached saturation with increasing substrate concentration. By fitting the data to the Michaelis-Menten equation, we obtained the kinetic parameters for their anti-oxidant nanozyme activity (**Table S1**). The K_m_ value reflects the affinity of the nanozyme material for the substrate; a lower K_m_ value indicates stronger substrate affinity and higher catalytic activity. The fitting data showed that, compared with CeO_2_, TC exhibited a 3-fold lower K_m_ value for CAT-like activity and a 1.35-fold lower K_m_ value for SOD-like activity. Furthermore, the catalytic efficiency constants (K_cat_/K_m_) for the CAT-like and SOD-like activities were enhanced by 7.7-fold and 7.2-fold, respectively, compared with those of CeO_2_. Furthermore, compared with the previously reported material, which exhibits CAT-like nanozyme activity (Res@PtZ-Z, Km = 95.54 mM) 56, the TC nanozyme heterojunction demonstrates a significantly lower K_m_ value (K_m_ = 5.7 mM). Taken together, these findings confirm the outstanding antioxidant nanozyme activity exhibited by the TC material. Collectively, these experimental findings provide critical validation for the exceptional ROS-scavenging capacity of TC, establishing a robust foundation for activating anti-oxidant stress pathways in subsequent bilayer microneedle applications.

### Characterization of the base layer of the hydrogel microneedles

**Figure 4:**
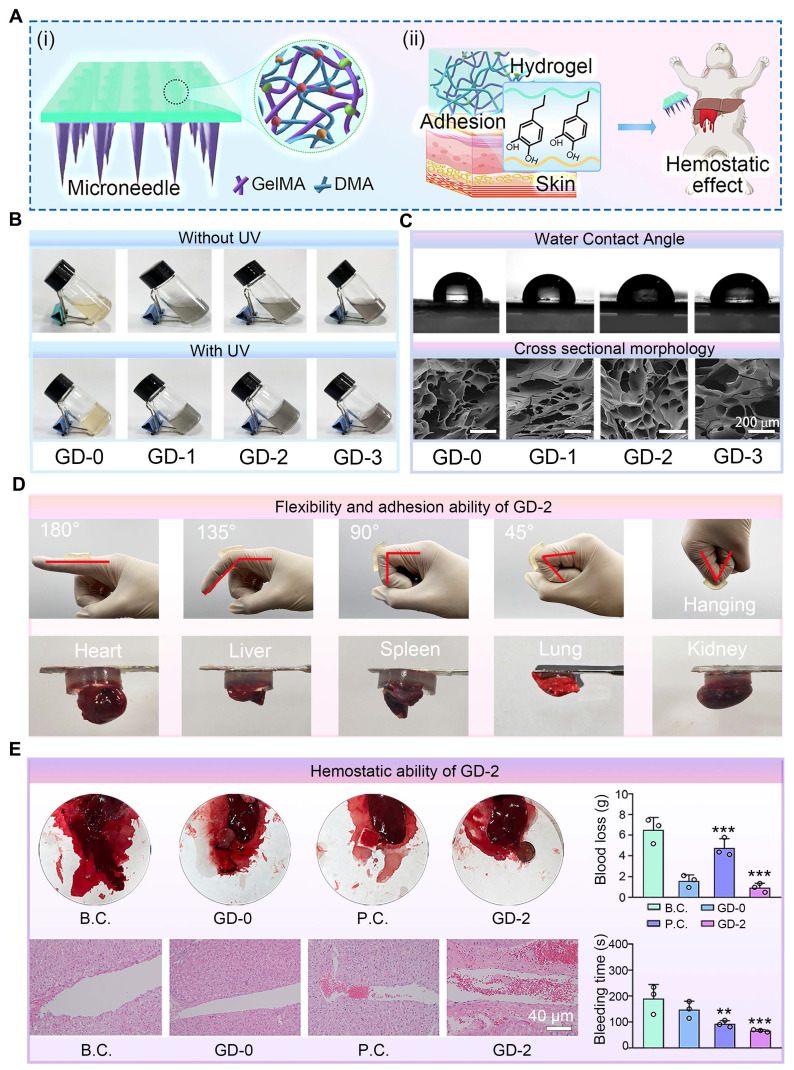
** Preparation and characterization of the adhesion and hemostatic base layers of the hydrogel microneedles.** (**A**) Diagram showing the composition and properties: (i) GelMA and DMA were chemically crosslinked to obtain a series of hydrogels; (ii) the hydrogels exhibited good tissue adhesion and noncompressive hemostatic effects; (**B**) Four kinds of hydrogels with various proportions of GelMA and DMA were prepared by UV crosslinking; (**C**) Images of the water contact angle and cross-sectional morphology of the freeze-dried hydrogels. Scale bar: 200 µm; (**D**) Evaluations of the flexibility and adhesion ability of the GD-2 hydrogel; (**E**) Evaluations of non-compressive hemostatic effect of the GD-2 hydrogels in a rabbit liver bleeding model. Optical images, H&E stained images of the bleeding tissue, and quantitative results of blood loss (n = 3) and bleeding time (n = 3) are showed. The values are expressed as the means ± SD. Compared with the blank control (B.C.) group, **P < 0.01, ***P < 0.001.

The wound repair process is highly orchestrated and intricate, encompassing four consecutive stages: hemostasis, inflammation, proliferation, and tissue remodeling 57. Vascular constriction and the activation of a series of clotting cascades following wound injury lead to wound hemostasis, which constitutes the body's first response to tissue damage 58. Timely hemostasis at the wound site is a critical step for accelerating healing and saving patients' lives. To endow bilayer microneedles with exceptional hemostatic properties, we developed a highly bioadhesive hydrogel composed of GelMA and methacrylated dopamine (DMA) as the base layer of the bilayer microneedles. This hydrogel could effectively adhere to the wound site to achieve hemostasis and accelerate wound healing (**Figure 4A**).

To systematically evaluate the impact of DMA incorporation on the physicochemical properties of the hydrogel base layer, we prepared composite hydrogels with varying DMA concentrations (GD-0, 1, 2, and 3) for comparative analysis. As shown in **Figure 4B**, the pure GelMA solution (GD-0) exhibited a pale-yellow color, whereas the solutions gradually darkened with increasing DMA content. Upon UV irradiation, all the solutions underwent a chemical crosslinking transition from liquid to solid, confirming the homogeneous dispersion of DMA within GelMA and its negligible interference with the gelation capability. The cross-sectional morphology and water contact angle data of the hydrogels (**Figure 4C** and **Figure S6**) revealed a unique porous microstructure, which facilitated the efficient absorption of wound exudate and maintenance of a moist wound microenvironment. The water contact angle results indicated enhanced hydrophilicity in the DMA-incorporated hydrogels, which was likely attributed to hydrogen bonding between water molecules and the amide/hydroxyl groups in DMA 59.

To further assess whether the mechanical properties meet clinical requirements, compression tests were conducted on GD-n hydrogels (**Figure S7**). The results demonstrated that DMA incorporation altered the mechanical behavior of the hydrogels, as DMA and GelMA interacted solely through physical crosslinking. Notably, excessive DMA loading (GD-3) significantly compromised compressive performance, which might hinder the application of the microneedles to skin wounds. Additionally, swelling tests (**Figure S8**) revealed that DMA incorporation did not adversely affect the water absorption capacity, likely because the enhanced hydrophilicity counteracted the reduced crosslinking density.

Based on these comprehensive physicochemical evaluations, the GD-2 hydrogel, which exhibited balanced overall performance, was selected for subsequent experiments.

### Evaluation of adhesive hemostasis performance

Tissue adhesion tests on joints and visceral organs were conducted to assess the adhesive properties of the GD hydrogel base layer. As demonstrated in **Figure 4D**, the GD hydrogels exhibited robust adhesion, successfully adhering to dynamic joints. Additionally, the hydrogel displayed sufficient toughness to deform without fracturing during joint flexion. Adhesion tests on diverse tissues, including the heart, liver, spleen, lung, and kidney, further confirmed the exceptional adhesive performance of the GD hydrogel. We further conducted corresponding quantitative measurements on the adhesive properties of the GD hydrogel using fresh porcine skin. The results, as shown in **Figure S9**, indicate a strong correlation between the adhesive strength of the GD hydrogel and the DMA content. The GD-0 hydrogel, which lacks DMA, exhibited very low adhesive force. As the DMA content increased, the adhesive strength gradually improved, with the GD-2 formulation achieving the highest adhesive force of approximately 2.3 N. Notably, a further increase in DMA content (GD-3) did not yield a statistically significant enhancement in adhesion compared to GD-2. These data collectively confirm the excellent adhesive performance of the GD hydrogel.

A female New Zealand rabbit liver puncture model was employed to evaluate the hemostatic efficacy of the GD hydrogel. As shown in **Figure 4E**, both the positive control (P.C.) group and the GD-2 group presented significantly shorter hemostasis times than the blank group did, with the GD-2 group achieving the shortest duration. Blood loss followed a similar trend, decreasing sharply from 6.51 ± 1.20 g in the blank group to 0.93 ± 0.15 g in the GD-2 group.

To further characterize the hemostatic mechanism, H&E histological staining of the liver puncture sites was performed. The results revealed that the GD hydrogel-treated wounds were densely populated with blood cells, which were effectively trapped at the wound interface. These findings suggest that the GD hydrogel not only provides a physical barrier for liver wounds but also enhances coagulation through bioactive components such as amino and polypeptide groups 60,61, thereby accelerating hemostasis and minimizing blood loss.

Collectively, these experimental findings critically validate the adhesive and hemostatic capabilities of the GD hydrogel, establishing a robust foundation for its application in bilayer microneedle systems.

### Characterization of the photothermal effect

The photothermal properties of Ti_3_C_2_ nanosheets and TC heterojunctions were characterized under 808 nm NIR laser irradiation at 2.5 W/cm², both Ti_3_C_2_ and TC materials exhibited pronounced photothermal effects, albeit with distinct heating profiles. Ti_3_C_2_ showed a gradual temperature increase from room temperature, reaching approximately 35 °C after 2 min of irradiation. The heating rate slightly increased thereafter, with the temperature rising to approximately 40 °C at 5 min, yielding an overall temperature elevation of approximately 20 °C.

In contrast, TC demonstrated a more rapid and pronounced photothermal response under identical irradiation conditions. Starting from approximately 28 °C, TC reached 37 °C within the first 2 min and continued to rise to approximately 45 °C at 5 min, which was about 5 °C higher than that of Ti_3_C_2_ at the same time point. The heating curve of TC was notably steeper, indicating superior photothermal conversion efficiency and faster thermal responsiveness (**Figure S10**). Based on the single heating-cooling curve and the corresponding thermal time constant, the photothermal conversion efficiency (η) of TC was determined to be 47.66% (**Figure S11**). This value is significantly superior to those previously reported for MXene-based nanozymes 62,63.

The enhanced photothermal performance of TC can be attributed to the construction of the Ti_3_C_2_/CeO_2_ heterojunction, which effectively enhances NIR absorption and facilitates efficient heat accumulation and transfer.

**Figure 5:**
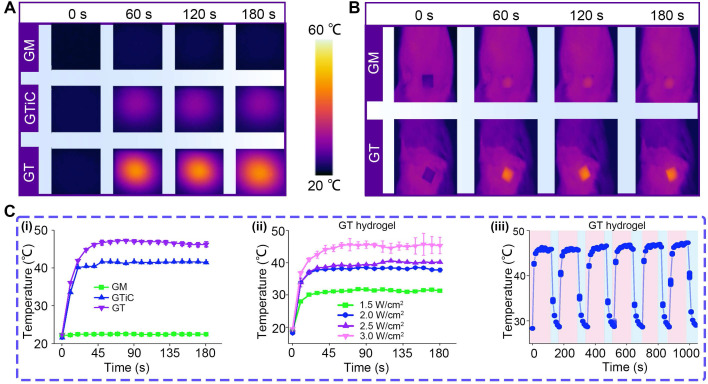
**Photothermal properties of nanoparticle-laden hydrogels.** (**A**) Thermal imaging of GM, GTiC, and GT hydrogels under 808 nm NIR laser irradiation (3.0 W/cm^2^) *in vitro*; (**B**) Thermal imaging of GM and GT hydrogels under 808 nm NIR laser irradiation (3.0 W/cm^2^) *in vivo*; (**C**) Photothermal heating curves: (i) temperature-time profiles of GM, GTiC, and GT hydrogels at a constant power density of 3.0 W/cm^2^; (ii) temperature profiles of GT under varying power densities (1.5, 2.0, 2.5, and 3.0 W/cm^2^) (n = 3), the values are expressed as the means ± SD; (iii) photothermal cycling stability of GT over six consecutive NIR on/off cycles at 3.0 W/cm^2^.

For the photothermal effects of the nanoparticle-laden hydrogels,** Figure 5A** and **B** present the thermal images of GM, GTiC, and GT hydrogels under NIR irradiation *in vitro* and *in vivo*, respectively, showing the surface temperature distribution over 0-180 s. GT exhibited significantly higher temperatures than GM and GTiC, indicating superior photothermal conversion performance.

**Figure 5C** (i) shows the temperature-time curves of the three hydrogels at a constant power density of 3.0 W/cm^2^. GT rapidly reached about 45 °C within 45 s and maintained this temperature, whereas GTiC only reached about 40 °C under identical conditions, demonstrating the superior photothermal responsiveness of GT. **Figure 5C** (ii) displays the temperature profiles of GT under varying power densities (1.5, 2.0, 2.5, and 3.0 W/cm^2^). With increasing power density, the temperature elevation of GT progressively increased. After 30 s of irradiation, the equilibrium temperatures reached approximately 30, 37, 38, and 45 ℃, respectively, exhibiting a distinct power-dependent photothermal conversion behavior. **Figure 5C** (iii) illustrates the temperature variation of GT over six NIR on/off cycles. Under 3.0 W/cm² irradiation, GT rapidly heated to 45 ℃ and quickly cooled to approximately 30 ℃ upon termination of irradiation. The temperature response remained consistent throughout six cycles, confirming excellent photothermal cycling stability.

Collectively, these results demonstrate that the TC heterojunction exhibits outstanding thermal responsiveness under NIR excitation, which can effectively enhance enzymatic reaction efficiency. Furthermore, previous studies have shown that TC can electrostatically capture bacteria in the acidic microenvironment associated with bacterial infection 22. This property, combined with its photothermal effect, confers remarkable antibacterial activity capable of eradicating drug-resistant bacteria and disrupting bacterial biofilms.

### Characterization of the bilayer microneedles (GTM hydrogels)

**Figure 6:**
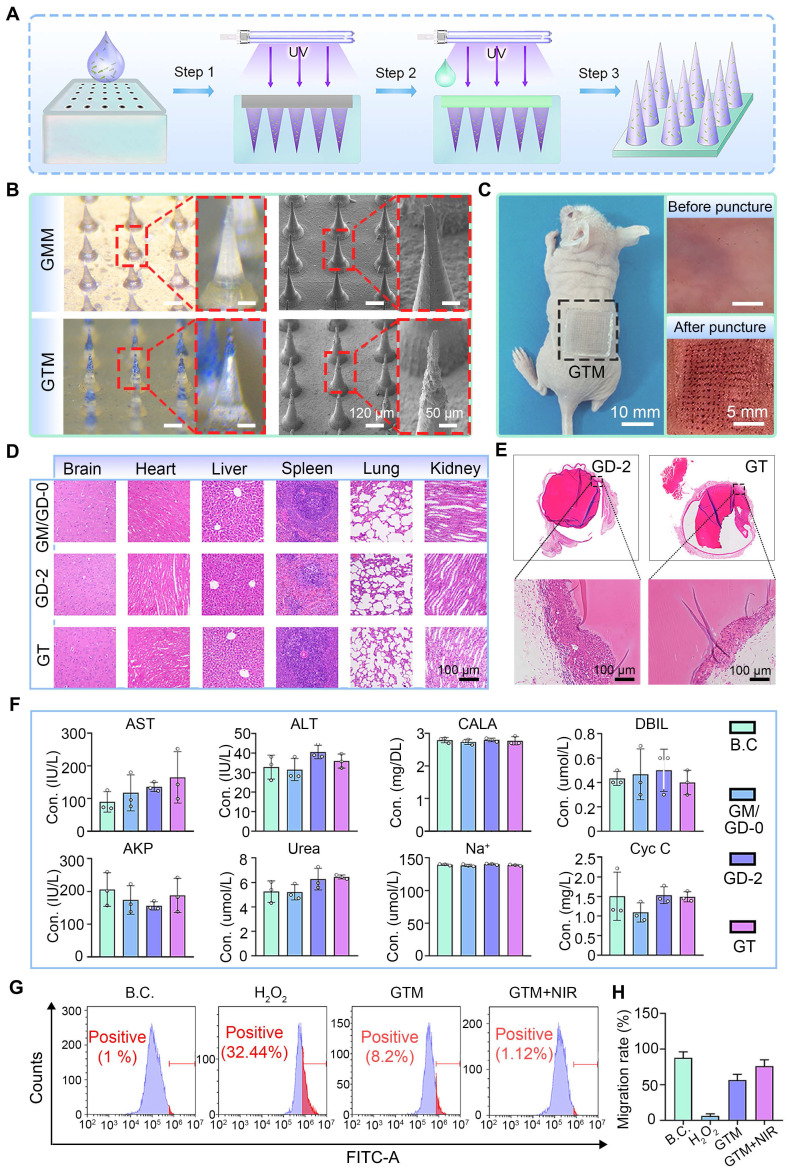
** Preparation and characterization of double-layered hydrogel microneedles.** (**A**) Diagram showing the preparation process of the double-layered hydrogel microneedles. Step 1: Adding the GT hydrogel precursor; Step 2: Adding the GD-2 hydrogel precursor; Step 3: demolding; (**B**) Morphologies of two kinds of microneedles. Scale bars: 120 µm and 50 μm; (**C**) GTM microneedles can puncture the skin of BALB/c nude mice. Scale bars: 10 mm and 5 mm; (**D**) H&E staining images of the organs. Scale bar: 100 µm; (**E**) H&E staining images of the fibrous capsule. Scale bar: 100 µm; (**F**) Blood biochemical tests (n = 3); (**G**) Anti-oxidative test by flow cytometry (n = 3); (**H**) Migration rate of L929 cells (n = 3). Values are expressed as means ± SD. For the blood biochemical tests, no significant differences were observed among all experimental groups. For the migration rate of L929 cells, statistical analysis revealed that all other experimental groups exhibited highly significant differences compared to the B.C. group.

Bilayer microneedles (GTM hydrogels) were fabricated via a casting method, where the GT hydrogel was utilized as the tip layer and the GD-2 hydrogel was used as the base layer (**Figure 6A**). For comparative analysis, control microneedles (GMM) without TC incorporation were prepared using the same protocol (**Figure 6B**). A distinct bilayer structure was observed in GTM compared with GMM, and the surface morphology of individual GTM tips was found to be rougher, indicating the presence of TC nanosheets on the tips.

The mechanical performance of the microneedle tips was evaluated through compression tests. The compression curves for both microneedle types are shown in **Figure S12**. The tips of both microneedles exhibited satisfactory mechanical strength. The maximum force sustained by a single GMM microneedle tip was 0.19 N, whereas that for a single GTM microneedle tip was 0.12 N. Both values met the penetration threshold requirement of greater than 0.058 N per needle tip 64. The mechanical strength of the GTM was evaluated through skin penetration tests on BALB/c nude mice. As shown in **Figure 6C**, GTM successfully penetrated the skin without structural failure, confirming its suitability for clinical applications.

The degradation behavior of wound dressings is directly related to their functional stability during use. We tested the degradation performance of the microneedles. As shown in **Figure S13**, the GTM microneedles maintained excellent structural integrity throughout the initial critical phase of wound healing: the degradation rate remained below 5% during the first 6 days. Even after prolonged incubation, the degradation rate reached only approximately 14.63% by day 15. This slow and controlled degradation profile strongly supports the functional stability and reliable structural integrity of the GTM microneedle patch during the essential stages of wound repair.

To comprehensively assess biocompatibility, GTM microneedles were subcutaneously implanted in SD rats. After 15 days, the visceral tissues and fibrous capsules surrounding the implants were subjected to H&E staining. No significant pathological abnormalities were detected in the visceral tissues (**Figure 6D**), suggesting the absence of systemic toxicity. Fibrous capsule formation, a typical immune response to foreign materials, was observed around all the microneedle implants (**Figure 6E**). Blood biochemical analysis (**Figure 6F**) further revealed that hepatic and renal function indices in the SD rats remained within normal ranges, with no statistically significant differences compared with those of the sham-surgery B.C. group. These findings collectively demonstrate that GTM microneedles exhibit excellent biocompatibility and mechanical reliability, meeting the stringent criteria for clinical translation.

L929 and HUVEC cells were selected as the model system to evaluate the anti-oxidant stress capability of GTM microneedles under *in vitro* conditions. Oxidative stress is induced by H_2_O_2_ treatment to simulate a microenvironment characterized by excessive ROS production 65. The ability of GTM microneedles to scavenge intracellular ROS was quantified by flow cytometry. As shown in **Figure 6G** and **Figure S14**, GTM microneedles demonstrated significant ROS clearance efficiency in treated L929 cells. Under near-infrared (NIR) light stimulation, photogenerated charge carriers were produced in greater quantities, and their migration was facilitated by the interfacial effects of the TC nanosheets, which collectively enhanced the anti-oxidant nanozyme activity of the GTM microneedles. Notably, the clearance of ROS by the GTM microneedles enhanced cell migration towards HUVEC cells and L929 cells. As shown in **Figures 6H and S15-16**, the observed cell migration and viability under near-infrared (NIR) stimulation were better than those under non-stimulated conditions, suggesting that the GTM microneedles may promote cell migration at the wound site, thereby accelerating healing.

In summary, GTM microneedles exhibited ROS-responsive scavenging properties and induced cell migration, highlighting their potential to accelerate diabetic wound healing through dual anti-oxidant and pro-regenerative mechanisms.

### Evaluation of diabetic wound healing

**Figure 7:**
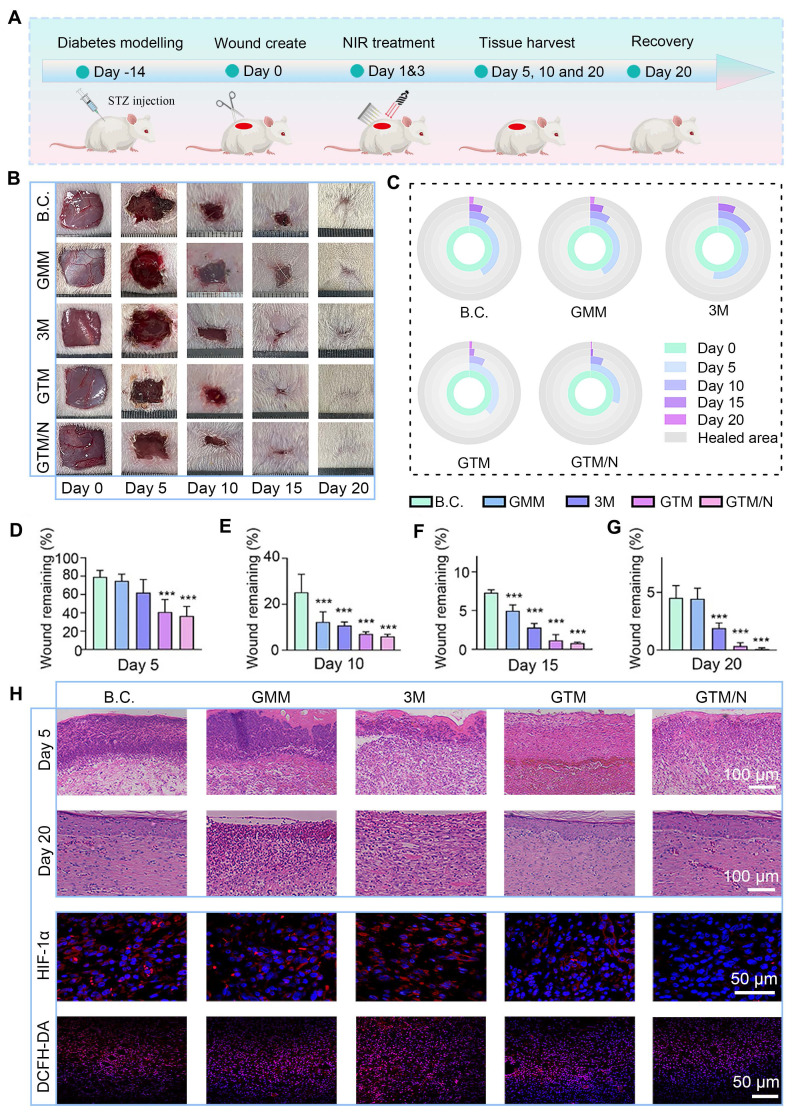
** Wound healing evaluations* in vivo*.** (**A**) Diagram showing the experimental protocols; (**B**) Images of the wound sites; (**C**) Dynamic traces of the wound area; (**D-G**) Wound remaining rate at each time point (n = 5); (**H**) Histological images of neoskin tissue, including H&E staining (scale bar: 100 µm) at Days 5 and 20, and fluorescence staining of HIF-a and DCFH-DA (scale bar: 50 µm) on Day 5. The values are expressed as the means ± SD. Compared with the blank control (B.C.) group, ***P < 0.001.

In this study, a full-thickness skin defect model was established in streptozotocin (STZ)-induced diabetic SD rats to evaluate the therapeutic efficacy of GTM microneedles on diabetic wounds. The experimental workflow is illustrated in **Figure 7A**. First, SD rats were intraperitoneally injected with STZ solution 66. After two weeks, stable hyperglycemia (blood glucose ≥15 mmol/L) was confirmed, indicating successful establishment of the diabetic model. A 1.5 cm × 1.5 cm full-thickness wound was created surgically on the dorsal skin of each rat.

The rats were randomly assigned to five groups: B.C. (blank control), GMM, 3M, GTM, and GTM/N, each receiving distinct treatments. The progression of wound healing across groups is shown in **Figure 7B** and **C**. The GTM/N group exhibited optimal wound closure, achieving complete re-epithelialization by Day 20, whereas the other groups showed residual unhealed areas. Quantitative analysis of the wound closure rates (**Figure 7D-G**) revealed that the GTM/N group consistently demonstrated the highest healing rate at all time points. By Day 20, the GTM/N group achieved a wound closure rate of 99.89% ± 0.07%, which significantly surpassed that of the B.C. group (95.48% ± 1.06%). The above results demonstrate that the photocatalytic therapeutic strategy delivered by GTM microneedles markedly facilitated wound repair in diabetic models.

Changes in animal body weight serve as a critical indicator for assessing the safety and tolerability of any treatment. Accordingly, we monitored the body weight of rats throughout the treatment period (**Figure S17**). The data demonstrate that none of the treatments, including GTM+NIR (GTM/N), resulted in any significant adverse effects on the overall health or body weight of the animals, supporting the favorable safety profile of our approach.

To further assess wound-healing quality, newly formed skin tissues were collected at early (Day 5), intermediate (Day 10), and late (Day 20) stages for histological staining. The H&E staining results are shown in **Figure 7H**. Granulation tissue was observed in all five groups to fill the skin defects during the early phase. Notably, the GTM/N group exhibited less inflammatory cell infiltration compared to the other groups. By Day 20, fully keratinized epithelium had formed in the GTM/N group, indicating superior healing quality, whereas incomplete healing persisted in the other groups. Further analysis of HIF-1α and DCFH-DA staining at the early stage of wound healing revealed that, owing to the excellent anti-oxidant stress activation of GTM under near-infrared light, the ROS content of the wound decreased significantly. While the ROS content decreased due to CAT-like enzyme activity, the generated oxygen significantly improved the hypoxic state of the wound, and the HIF-1α index also decreased significantly 67,68, which confirmed the effectiveness of GTM microneedles in the treatment of anti-oxidant stress.

**Figure 8:**
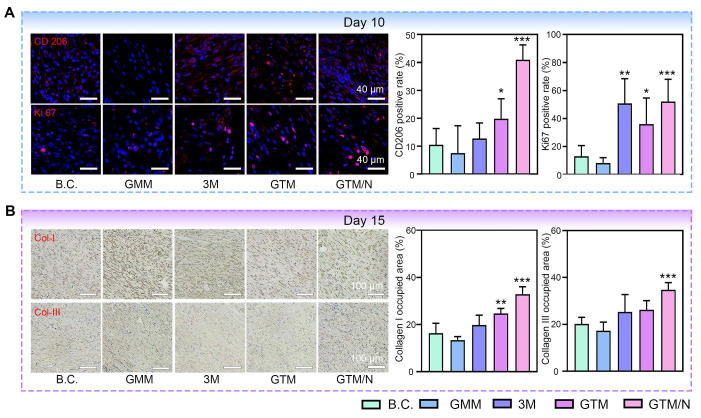
** GTM accelerated wound healing due to its multiple bioactive properties.** (**A**) Histological staining images and quantitative histological analysis of neoskin tissues at Day 10, including CD206 and Ki67. Scale bar: 40 μm; (**B**) Histological staining images and quantitative histological analysis of neoskin tissues at Day 15, including Col-I and Col-III. Scale bar: 100 μm. Values are expressed as the mean ± SD (n = 5). Compared with the blank control (B.C.) group, *P < 0.05, **P < 0.01, ***P < 0.001.

Mid-phase wound healing primarily involves proliferation and anti-inflammatory processes 69. CD206 and Ki67 immunofluorescence staining of mid-phase wound tissues (**Figure 8A**), revealed that GTM/N treatment accelerated the polarization of macrophages towards the M2 phenotype (marked by CD206), thereby reducing inflammation in the wound area and promoting healing. Concurrently, cell proliferation at the wound site (indicated by Ki-67) was significantly greater in the GTM/N group than in the B.C. group, further facilitating rapid tissue repair.

Late-phase wound healing is dominated by collagen remodeling 70. To evaluate this stage, we performed collagen immunohistochemical staining on late-phase wound tissues (**Figure 8B**). Based on quantitative analysis, the GTM/N group exhibited the highest level of collagen deposition compared to other groups. Notably, the type I collagen content significantly exceeded the type III collagen content. This phenomenon occurs because type III collagen is synthesized predominantly by proliferating fibroblasts during the early remodeling phase, whereas type I collagen becomes the dominant component in the late remodeling phase as type III collagen is gradually degraded 71. The predominance of type I collagen in the regenerated tissue closely resembles the composition of normal skin, indicating superior healing quality in the GTM/N group.

In summary, under the treatment of NIR-enhanced anti-oxidant stress, the GTM system accelerated diabetic wound repair through coordinated enhancement of anti-inflammatory responses, proliferative activity, and extracellular matrix remodeling at the wound site.

## Conclusions

In this study, a NIR-enhanced antioxidant nanozyme heterojunction hydrogel microneedle system (GTM) was successfully developed for the treatment of diabetic chronic wounds.

Ti_3_C_2_/CeO_2_ (TC) heterojunction nanozymes were synthesized via combined etching and solvothermal methods. The engineered Schottky heterojunction efficiently facilitated charge migration across the interface while inhibiting the recombination of photogenerated electron-hole pairs. DFT simulations and electrochemical analyses confirmed that the heterojunction structure reduced the bandgap from 2.44 eV (pristine CeO_2_) to 0.32 eV, significantly enhancing NIR absorption and photocatalytic performance. Under NIR irradiation, TC exhibited excellent SOD-like and CAT-like antioxidant activities, with catalytic efficiency constants (K_cat_/K_m_) enhanced by 7.7-fold and 7.2-fold, respectively, compared to pristine CeO_2_. These enhancements can be attributed to the synergistic effects of interfacial charge transfer, photogenerated electron-hole separation, and photothermal conversion. Additionally, TC demonstrated superior photothermal conversion performance under 808 nm NIR irradiation, reaching approximately 45 °C within 5 min at 2.5 W/cm^2^, which was about 5 °C higher than that of pristine Ti_3_C_2_. The TC-laden hydrogel (GT) exhibited power-dependent temperature modulation and excellent photothermal cycling stability over six consecutive on/off cycles, enabling enhanced enzymatic catalysis and mild photothermal therapy with antibacterial and anti-inflammatory effects.

A bilayer microneedle system was fabricated via a two-step photopolymerization process. The GelMA-DMA base layer exhibited enhanced hydrophilicity, appropriate compressive strength, and excellent wet-tissue adhesion. In a rabbit liver bleeding model, GD-2 achieved rapid hemostasis, reducing blood loss from 6.51 g (blank control) to 0.93 g, attributed to both physical barrier formation and bioactive coagulation promotion mediated by amino and polypeptide groups. The TC-loaded GelMA tip layer provided sustained NIR-enhanced antioxidant activity, demonstrating significant ROS-scavenging capacity and promoting cell migration in H_2_O_2_-stimulated L929 fibroblasts as well as HUVECs.

*In vivo* biocompatibility assessments in SD rats revealed no obvious fibrous capsule formation or organ toxicity after implantation, with blood biochemical indices remaining within normal ranges. In the STZ-induced diabetic SD rat full-thickness skin defect model, the GTM/N group (with NIR irradiation) achieved a wound closure rate of 99.89% ± 0.07% by Day 20, significantly superior to the blank control (95.48% ± 1.06%), GMM, and 3M commercial dressing groups. Histological analyses demonstrated that GTM/N treatment effectively reduced ROS levels and alleviated hypoxia, promoted M2 macrophage polarization, accelerated cell proliferation, and enhanced type I collagen deposition, collectively indicating superior wound healing quality with complete re-epithelialization.

In summary, the GTM bilayer microneedle system successfully integrates rapid hemostasis, NIR-enhanced antioxidant nanozyme activity, and photothermal therapeutic functions with excellent biocompatibility. This multifunctional platform provides a promising, clinically translatable strategy to address the challenges of excessive ROS accumulation, microvascular dysfunction, and persistent bleeding in diabetic chronic wounds, offering new insights into heterojunction nanozyme design and photocatalytic interventions for treating oxidative stress-related diseases.

## Supplementary Material

Supplementary methods, figures, and table.

## Figures and Tables

**Table 1:** Nomenclature, compositions, and processing parameters of GT-n hydrogels

	15% GelMA (g)	TC (mg)	10% I2959 (µL)	UV power (W)	Curing time (s)
GT-1	20	0	50	160	300
GT-2	20	10	50	160	300
GT-3	20	20	50	160	300

**Table 2:** Nomenclature, compositions, and processing parameters of GD-n hydrogels

	15% GelMA (g)	10% DMA (g)	10% I2959 (µL)	UV power (W)	Curing time (s)
GD-0	20	0	50	160	300
GD-1	20	10	50	160	300
GD-2	20	20	50	160	300
GD-3	20	80	50	160	300

## Data Availability

The data that support the findings of this study are available from the corresponding author upon reasonable request.

## Abbreviations

B.C.: Blank control
CAT: catalase
DFT: density functional theory
DMA: methacrylated dopamine
DRS: diffuse reflectance spectroscopy
EDS: energy-dispersive X-ray spectroscopy
EIS: Electrochemical impedance spectroscopy
ESR: Electron paramagnetic resonance
Gce: GelMA-CeO_2_ hydrogel
GD: GelMA-DMA composite hydrogels
GGA: generalized gradient approximation
GM: GelMA hydrogel
GMM: neat GelMA microneedles with both the needle tip and base composed of GelMA
GPx: glutathione peroxidase
GOx: glucose oxidase
GT: GelMA-TC heterojunction composite hydrogels
GTiC: GelMA-Ti_3_C_2_ hydrogel
GTM: microneedles of GT components form the needle tip and GD components form the base
GTM/N: GTM microneedles combined with NIR-mediated photothermal therapy
I2959: Irgacure 2959
LSPR: localized surface plasmon resonance
LSV: linear sweep voltammetry
Met: methionine solution
NBT: nitroblue tetrazolium
NIR: near-infrared
P.C.: Positive control
PAW: projected augmented wave
PBE: Perdew-Burke-Ernzerhof
PDA: polydopamine
PDMS: polydimethylsiloxane
PL: photoluminescence
PM: PDA/MnO_2_ composite material
POD: peroxidase
ROS: reactive oxygen species
SEM: Scanning electron microscope
SOD: superoxide dismutase
SPF: specific-pathogen-free
TC: Ti_3_C_2_/CeO_2_
XPS: X-ray photoelectron spectroscopy
XRD: X-ray diffraction
