# Supplementary material for: Bioadhesive and hemostatic microneedles integrated with NIR-responsive Ti3C2/CeO2 heterojunction nanozymes for accelerated diabetic wound healing
Source: Theranostics. 2026 Mar 9;16(10):5296–318. doi: 10.7150/thno.130627 (PMC13080654; doi:10.7150/thno.130627)
Supplement: Supplementary file 1 — Supplementary methods, figures, and table. [file thnov16p5296s1.pdf]

Supplementary Information

Bioadhesive and hemostatic microneedles integrated with NIR-responsive Ti_3C_2/CeO_2 heterojunction nanozymes for accelerated diabetic wound healing

Zesheng Chen[a,b,c,1], Zhengyao Zhang[d,1], Lingfeng Tu[d,e,1], Guanyi Wang[c], Wang Wang[c], Ziqiang Xu[b], Feng Yu[a], Yinsuo Jia[a], Zijian Wang[c,*], Kanghong Hu[a,*] and Weikang Hu[a,b,*]

[a] Stem Cells and Tissue Engineering Manufacture Center, School of Life Sciences, Hubei University, Wuhan 430062, China

[b] Hubei Province Key Laboratory of Biotechnology of Chinese Traditional Medicine, College of Health Science and Engineering, Hubei University, Wuhan 430062, China

[c] Department of Urology, Hubei Key Laboratory of Urological Diseases, Cancer Precision Diagnosis and Treatment and Translational Medicine Hubei Engineering Research Center, Zhongnan Hospital of Wuhan University, Wuhan 430071, China

[d] Department of Biomedical Engineering, Hubei Province Key Laboratory of Allergy and Immune Related Disease, TaiKang Medical School (School of Basic Medical Sciences), Wuhan University, Wuhan 430071, China

[e] Department of Nuclear Medicine, Chongqing Hospital, Union Hospital, Tongji Medical College, Huazhong University of Science and Technology, 799 Liangjiang Avenue, Yubei District, Chongqing 401121, China

[1] These authors contributed equally to this work.

* Corresponding authors at: huwkang@hubu.edu.cn (W. Hu); hukanghong@hubu.edu.cn (K. Hu); zijianwang@whu.edu.cn (Z. Wang)

Experimental Methods

SEM observation

The morphology of TC heterojunction and composite biomaterials were observed using a scanning electron microscopy (SEM, AMBER, TESCAN, Czech) and energy-dispersive X-ray spectroscopy (EDS, ChemiSEM, Thermo-Fisher, USA).

XRD analysis

The chemical structure of the sample was characterized and analyzed by X-ray diffraction (XRD, D8A25, Bruker, Germany). For the XRD test, Cu Kα was used as a monochromatic light diffraction source to collect diffraction data of samples in the 2θ range of 5°–70° at a scanning rate of 10 ° min^{-1}.

XPS analysis

The chemical structure of the sample was characterized and analyzed by X-ray photoelectron spectroscopy (XPS, EscaLab 250Xi, Scientic Thermofisher, USA).

UV-Vis analysis

A UV spectrophotometer (UV–vis, UV3600, SHIMADZU, Japan) was used to analyze the light absorption efficiency and nanoenzyme-like activity of samples.

Compression test

The hydrogel samples were shaped into cylindrical specimens (height: 0.5 cm, diameter: 1.5 cm). Compression measurements were performed on an HC-CL100N universal testing machine at a constant compression rate of 0.1 cm min^{-1}. Throughout the testing process, the deformation behavior of the hydrogel was visually documented using a digital camera. The elastic modulus was derived from the initial linear region of the stress–strain curve (strain < 10%), while the fracture energy was obtained by integrating the stress–strain curve from the onset of loading to the point of fracture.

Water contact angle test

Hydrogel films were prepared using a mold and allowed to dry under ambient conditions at room temperature. The surface wettability of the resulting films was characterized using an SZ-CAMB1 contact angle analyzer, and the contact angle values were determined via the sessile drop measurement technique.

Swelling test

The hydrogel was cut into 0.5cm, 0.5cm, and 0.5cm cubes, and vacuum dried at 60 °C for 24h to remove the water in the hydrogel. The initial dry weight of each hydrogel specimen was determined using an analytical balance. The samples were subsequently submerged in PBS buffer solution. At predetermined time intervals, the hydrogels were retrieved, and residual surface moisture was gently blotted with filter paper before reweighing on the analytical balance to record the swollen mass.

Degradation Rate

Hydrogel samples were cut into cubes measuring 0.5 cm × 0.5 cm × 0.5 cm. Four experimental groups were established, with each group consisting of 24 parallel samples. Each sample was immersed in 10 mL of PBS solution and placed under a constant temperature shaker set at 37 °C. Degradation was observed and recorded on days 0, 1, 2, 4, 6, 8, 10, 12 and 15. Three parallel samples from each group were weighed at each time point, and the degradation rate was calculated. The degradation rate (Weight loss) is calculated as follows:

$$Degradation\,rate\ (\%) = \frac{(W_i - W_t)}{W_t} \times 100\%$$

where the W_i indicates the initial weight of each experimental sample, and the W_t indicates the instant weight of that at a predetermined time.

Adhesion test

The tissue adhesion capability of the hydrogel was evaluated using fresh porcine skin specimens in conjunction with a universal mechanical testing machine. The porcine skin was freshly harvested and the underlying adipose tissue was carefully excised. The cleaned skin was then trimmed into rectangular strips of 30 mm × 50 mm. GD hydrogel samples were molded into disc-shaped specimens with dimensions of 15 mm in diameter and 1 mm in thickness. One strip of porcine skin (with the dermis layer facing upward) was horizontally fixed onto a rigid plate attached to the lower clamp of the testing machine. A GD hydrogel disc was placed at the center of the skin, and another strip of porcine skin (with the dermis layer facing downward) was aligned and placed over the hydrogel. The assembly was pressed firmly for 30 seconds to ensure full

contact between the hydrogel and the skin interfaces and to remove excess air. Using the universal material testing machine, the maximum force required to peel the hydrogel from the porcine skin was measured at a tensile speed of 1 mm/min.

Flow cytometry

L929 cells were seeded into the lower chamber of Tran-swell plate. The experimental group consisted of blank control group (b.c.), H_2O_2 treatment group (p.c.), GTM group, GTM+NIR group (the upper cavity added GTM and near-infrared irradiation). All the treated cells were incubated with DCFH-DA dye at 37 ° C for 30 minutes. After washing with serum-free medium for three times, the intracellular ROS content was detected by flow cytometry (ACS AriaTM Fusion, BD, USA).

Evaluation of Antioxidant Stress Performance

To systematically evaluate the antioxidant stress resistance of microneedles, L929 and HUVEC cells were used as cell models, the treated cells are collected and densely seeded on 6-well plates. When the cells reach a confluent monolayer, use a sterile pipette tip to create an artificial "scratch" area on the monolayer of cells at a certain pressure and speed. The "scratched" state was photographed using an inverted fluorescence microscope at 0 h and 48 h to assess the cell migration process.

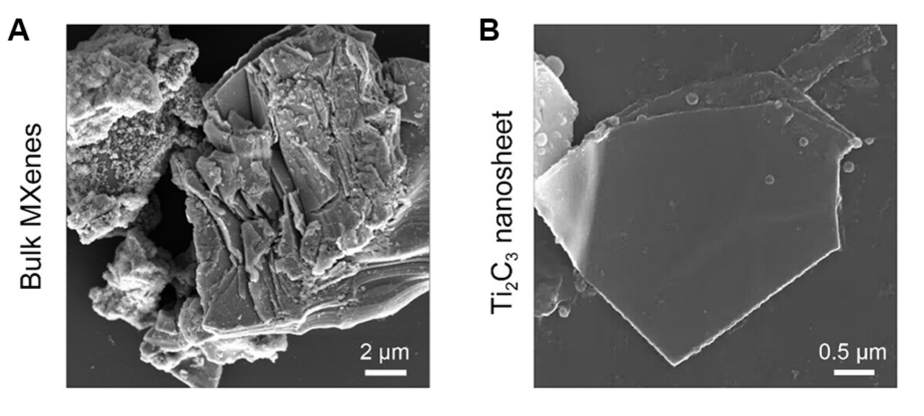

Figure S1. SEM images of bulk MXenes and monolayer Ti_3C_2 nanosheets. Scale bar: 2 μm and 0.5 μm.

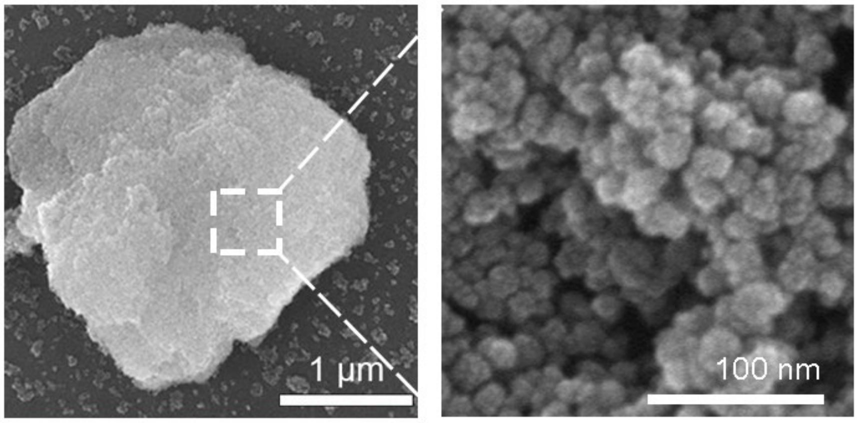

Figure S2. SEM images of TC, Scale bar: 1 μm and 100 nm.

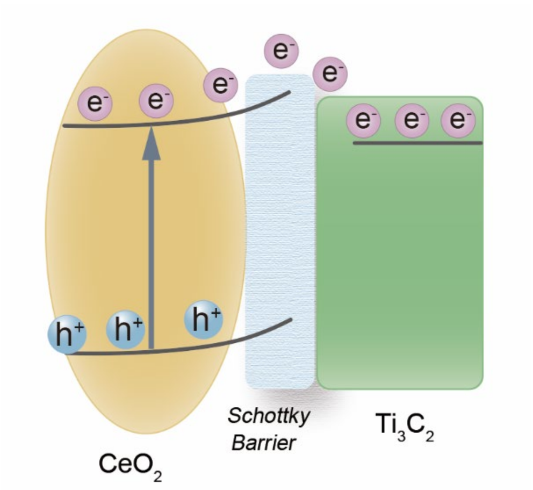

Figure S3. The energy band structure diagram of TC.

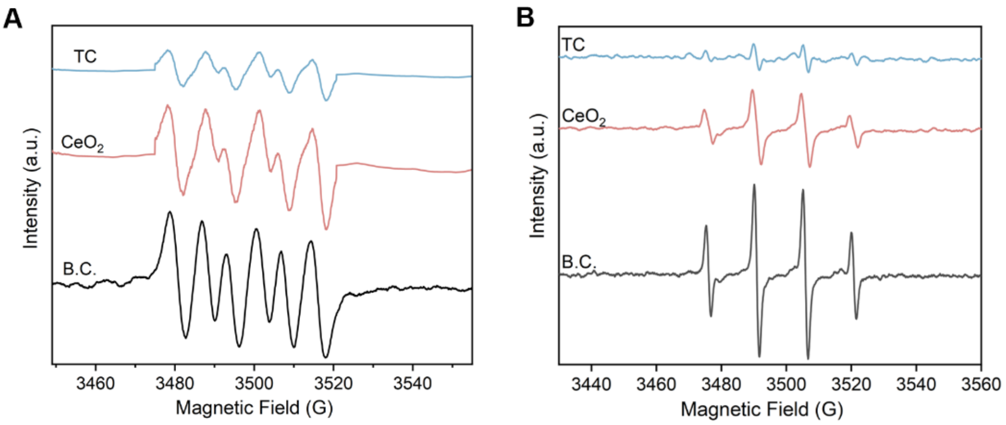

Figure S4. EPR spectra for (A) $\cdot OH$ and (B) $\cdot O_2^-$ under differing reaction conditions.

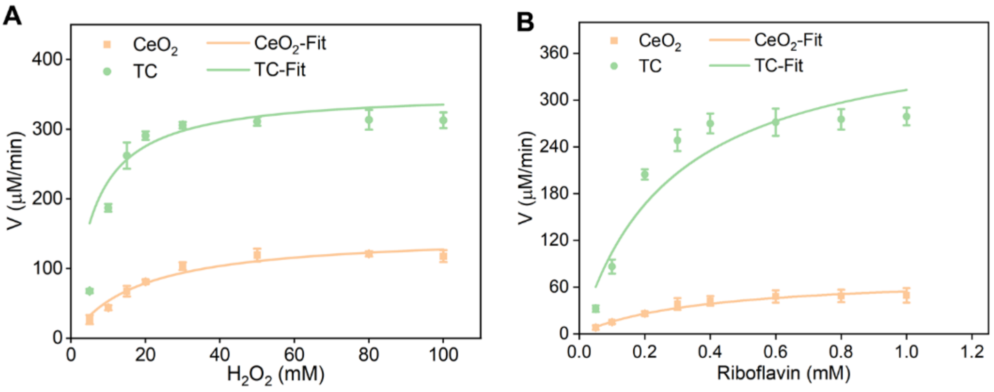

Figure S5. The Michaelis–Menten at various concentrations of (A) H_2O_2 and (B) Riboflavin. Values are expressed as the mean ± SD.

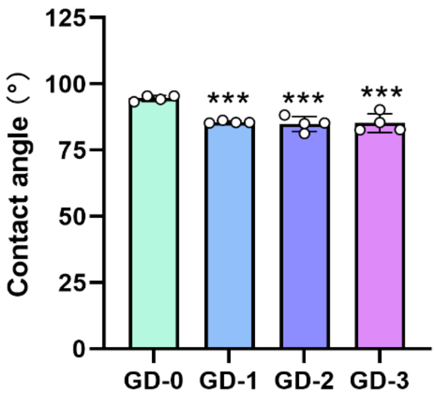

Figure S6. Quantitative results of water contact angle of GD-n hydrogels (n = 4). Values are expressed as the mean ± SD. Compared to GD-0 group, ***$P < 0.001$

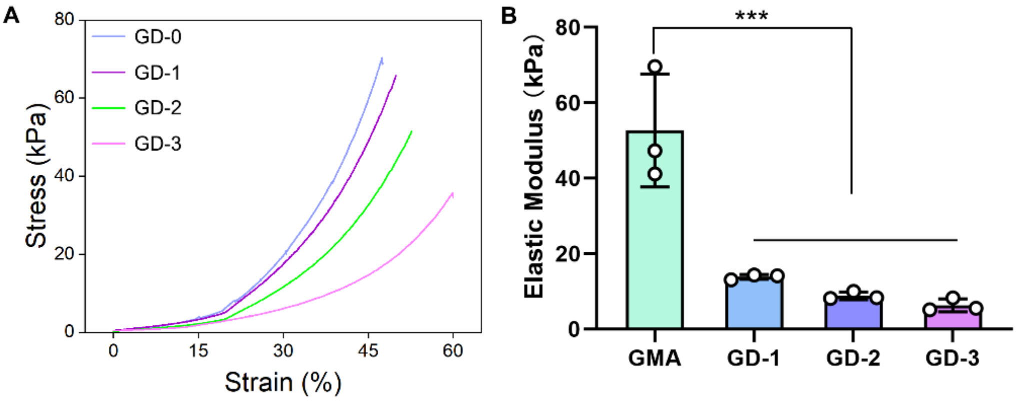

Figure S7. Compression test of GD-n hydrogels. (A) Stress-strain curves; (B) Elastic modulus (n = 3). Values are expressed as the mean ± SD. Compared to GD-0 group, ***$P < 0.001$.

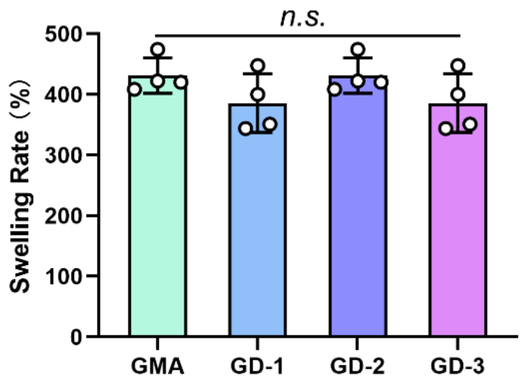

Figure S8. Equilibrium swelling ratio of dried GD-n hydrogels (n = 3). Values are expressed as the mean ± SD. For inter-groups comparison, *n.s.* indicated no significance.

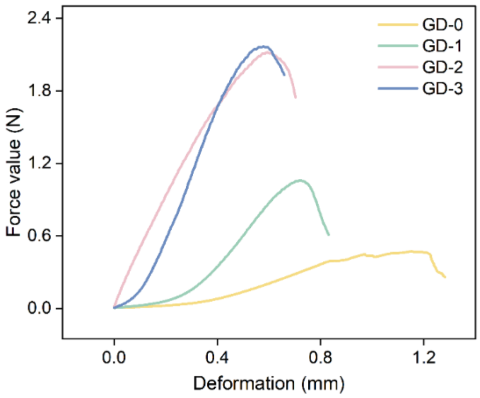

Figure S9. The adhesion force of GD-n hydrogel.

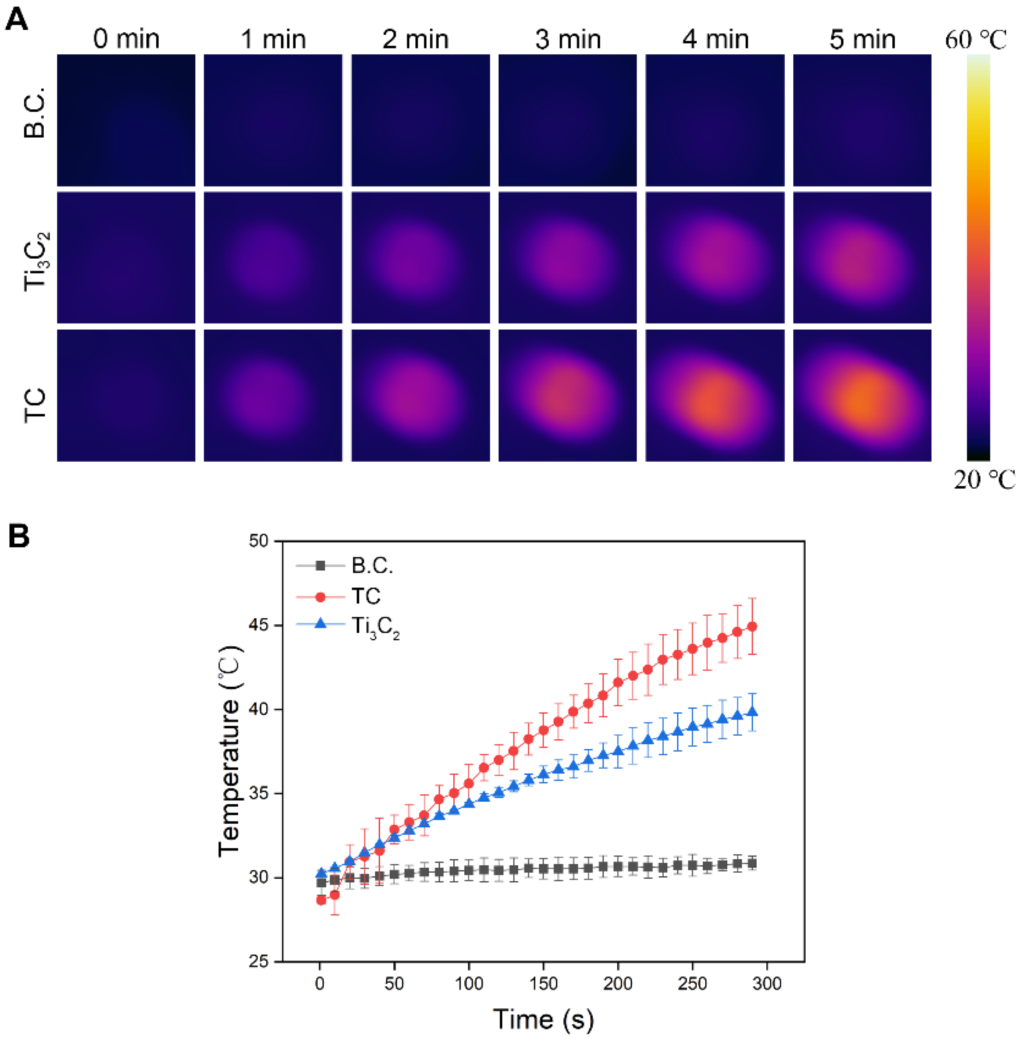

Figure S10. The photothermal effect of Ti_3C_2 and TC (n = 3). Values are expressed as the mean ± SD.

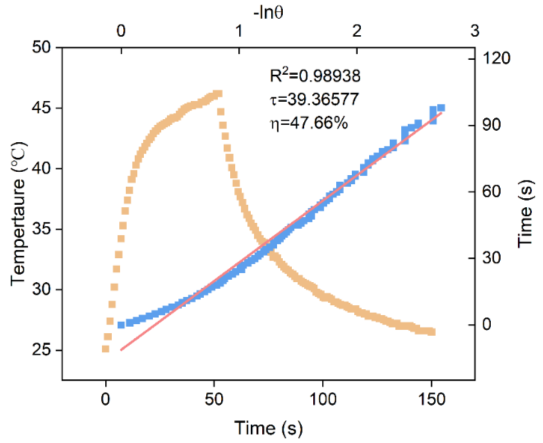

Figure S11. The fitting curve of the photothermal conversion efficiency of TC.

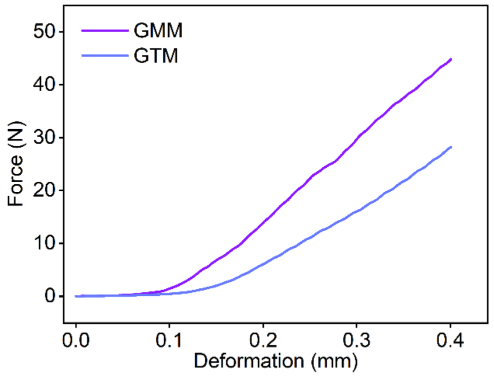

Figure S12. The compressive mechanical curve of microneedles.

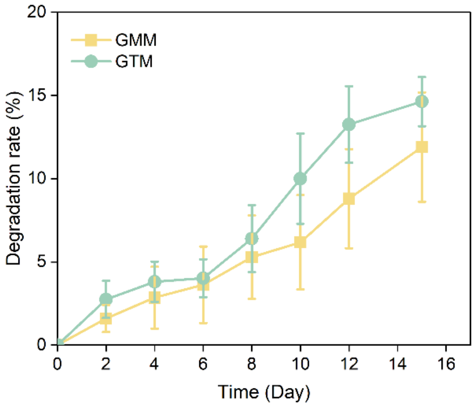

Figure S13. The degradation rates of GMM and GTM (n=3). Values are expressed as the mean ± SD.

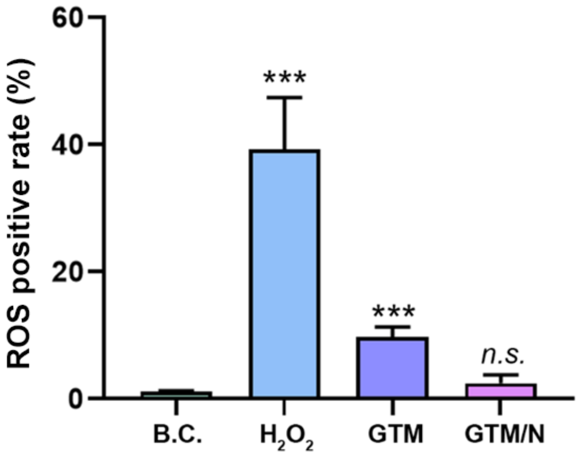

Figure S14. ROS positive rate of L929 cells (n = 3, biological replicates). Values are expressed as the mean ± SD. Compared to B.C. group, *n.s.* indicated no significance, ****P* < 0.001.

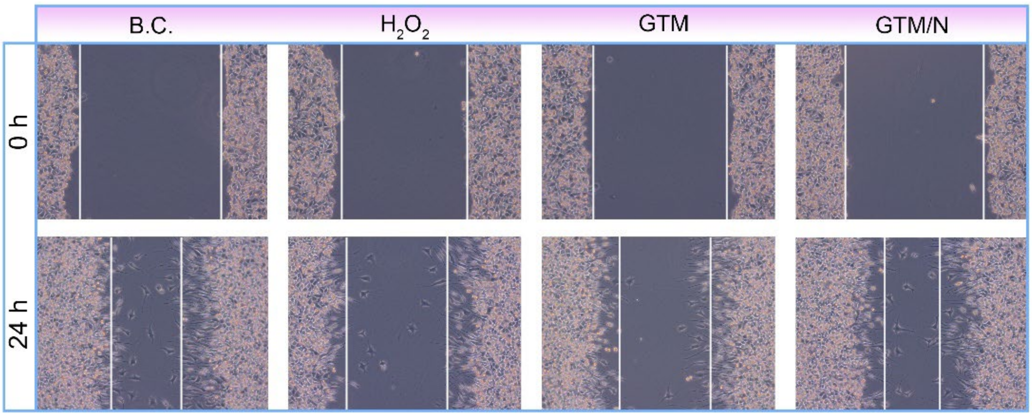

Figure S15. Images of L929 cells migration assay at varied time point.

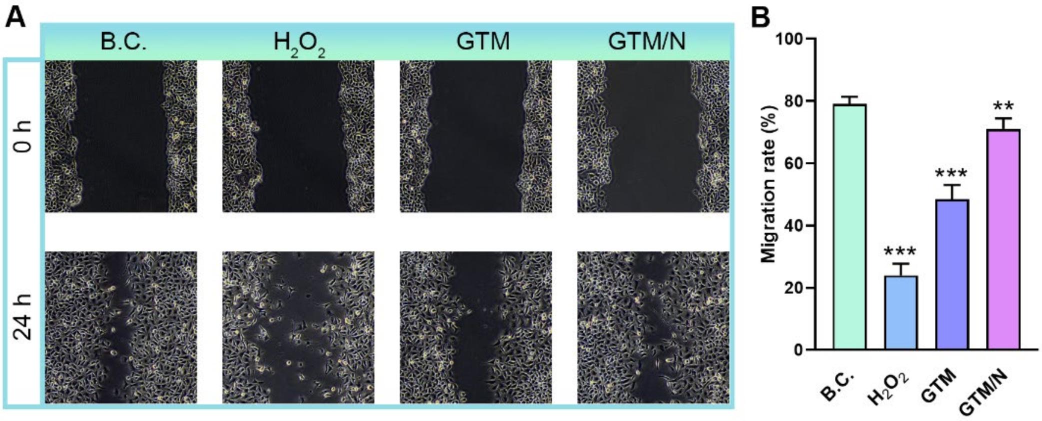

Figure S16. Images (A) and migration rate (B) of HUVEC cells migration assay. Values are expressed as the mean ± SD. Compared to the B.C. group, $^{**}P < 0.01$, $^{***}P < 0.001$.

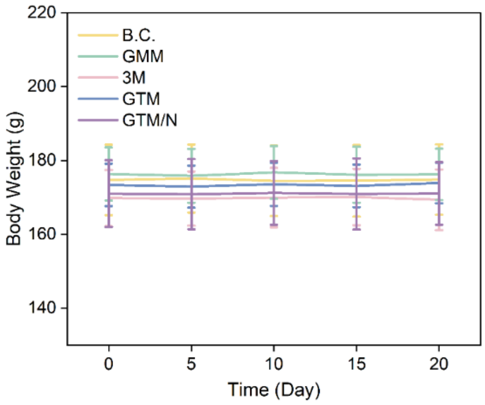

Figure S17. The body weight of diabetic rats during the experimental process (n = 5).Values are expressed as the mean ± SD.

Table S1 Kinetic parameters of different nanozymes.

Nanozyme	Substrates	V_{max} $\mu M \cdot min^{-1}$	K_m mM	K_{cat} min^{-1}	K_{cat}/K_m $mM^{-1} \cdot min^{-1}$
CeO_2	H_2O_2	150.86	18.06	301.72	16.22
	Riboflavin	75.37	0.38	150.74	396.68
TC	H_2O_2	354.9	5.7	709.8	124.53
	Riboflavin	401.97	0.28	803.94	2871.21
